# Suicide prevention training for allied health professionals within healthcare environments: A scoping review

**DOI:** 10.1371/journal.pone.0326738

**Published:** 2025-08-08

**Authors:** Jade Rentzoulis, Amanda Gilmore, Rowena Saheb, Arianne C. Reis

**Affiliations:** 1 School of Health Sciences, Western Sydney University, Campbelltown, New South Wales, Australia; 2 Mental Health Services, Western Sydney Local Health District, Cumberland, New South Wales, Australia; 3 Translational Health Research Institute, Western Sydney University, Campbelltown, New South Wales, Australia; Universidade Federal do Rio Grande do Sul, BRAZIL

## Abstract

Despite the knowledge that many individuals who die by suicide have contact with a health professional a short time prior to their death, the delivery and quality of clinical care is reportedly variable. Those affected by suicide have reported poor management of suicidal behavior, noting that many health professionals lack an understanding of, and carry negative attitudes toward suicidality. Though quality crisis care and follow-up treatment have shown to reduce the frequency of repeated suicide attempts, organizational barriers and lack of appropriate training mean these are not always provided. This review aimed to synthesize the evidence on suicide prevention training delivered to allied health professionals within healthcare environments. A scoping review of empirical research on the topic was undertaken using the JBI methodological framework, followed by a content synthesis of the evidence to explore the key characteristics of training and their outcomes. The databases included in the search were MEDLINE, CINAHL, PSYCHinfo, Scopus, and Embase. Final search and extraction from all databases were conducted on the same day in April 2021 and repeated in July 2023. The extracted data was collated and presented in tabular form to facilitate analysis. The review identified 33 relevant articles. The evidence base was largely heterogenous, involving a wide range of training types and underpinning frameworks. The measurement tools used to assess effectiveness of training were mostly not validated, with predominant use of short-term pre-post measures frequently based on perceptions of change rather than objective measures of behavior change. Significantly, evidence presented was generally weak and inconsistent between studies. This review highlighted significant gaps in current knowledge within the field of suicide prevention training delivered to allied health professionals. Investing in efficacious training and professional development opportunities is key to building the capacity to reduce suicide mortality in our community.

## Introduction

Suicide is recognized as one of the top leading causes of death globally [1,2], with suicide rates steadily increasing around the world in the last twenty years [2]. The World Health Organization (WHO) have prioritized the reduction of suicide mortality by 2030, noting the need for a collaborative approach across the health sector and government bodies to achieve this goal. In response to WHO’s call, 38 countries have adopted national prevention strategies implementing varied methods to reduce the number of suicides, often with recommendations for additional education in healthcare [3].

It is generally assumed that those who work in the field of mental health have greater exposure to people at risk of suicide compared with other health professions; however, research suggests that this is not the case [4]. In recent years, it has been highlighted that many individuals experiencing suicidality come into contact with general health care services in the 12 months prior to taking their own lives, with a contact rate average of 44% in the 1 month prior [5]. Yet the delivery and quality of clinical care is reportedly variable [6]. Study participants have reported poor management of their suicidal behavior, noting that many health professionals lack an understanding of, and carry negative attitudes toward suicidality. Participants also report inconsistent follow-up post-suicide crisis (aftercare), while others have experienced violent or punitive care, often resulting in avoidance of future help-seeking [7,8]. Though quality crisis care and follow-up treatment have shown to reduce the frequency of repeated suicide attempts, organizational barriers and lack of appropriate training mean these are not always provided [9].

While there is no universally accepted definition of allied health, Allied Health Professions Australia state that allied health professions are all health-related professions outside of medical, nursing and dentistry [10]. Allied health professionals (AHPs) are required to maintain and update their skills and knowledge throughout their career by participating in continuing professional development (CPD) and/or in-service training regularly [11]. This provides the necessary elements for increased competence and professional growth, particularly as ongoing developments in their field of practice emerge [12,13].

Suicide prevention training is one of a vast number of CPD courses available to AHP’s globally. Unfortunately, a significant number of health professionals, including AHPs, report a lack of specialized training to support people at risk of suicide as one of the most challenging areas of daily practice [14]. For example, an Italian study discussed how 88% of their study participants reported never receiving suicide assessment or intervention training [15], while a study in the United States showed that suicide prevention training is only mandated for health professionals in 10 out of 50 states, and that it is generally targeted at those working in mental or behavioral health [16], therefore not including a range of AHPs.

The evidence surrounding the positive outcomes of suicide prevention training for health professionals is vast; however, it is acknowledged that the availability and delivery of such training is inconsistent [17]. Many different types of evidence-based suicide prevention training exist and are delivered around the world, including gatekeeper or means-restriction training, strengths-focused and applied skills training [17,18]. There are also various modes of delivery for such training including in-person workshops, online self-directed modules, and blended learning [19]. Training in suicide prevention intends to provide the skills and knowledge to identify suicidal behaviors, conduct effective risk assessment, provide treatment and support, and connect at-risk individuals with necessary services [20].

Despite the number of suicide prevention training options available to health professionals [17,18], it is currently unclear at what rate this is being delivered specifically to AHPs and how this may differ between allied health disciplines. Prior to commencing this review, a preliminary search was undertaken to identify and assess the volume of existing literature addressing suicide prevention training for AHPs. Several reviews were located examining suicide prevention interventions and factors influencing suicide risk assessment [21–25]; however, no studies were found that specifically examined training in this context. To address this gap in knowledge and to inform further and more detailed inquiry into this important field of suicide prevention practice, this scoping review sought to identify the features of suicide prevention training AHPs working within the healthcare system receive, and the most common forms of delivery of this training, as well as the frameworks/models underpinning them.

### Review questions

i) What types of suicide prevention training have been delivered to allied health professionals in the last decade?
ii) Which allied health disciplines are targeted for suicide prevention training?
iii) What are the frameworks/models underpinning the training provided and what are the key constructs of the training content?
iv) How are such programs assessed for their effectiveness and what are the outcomes?

### Inclusion criteria

#### Participants.

To ensure a comprehensive search, all health professions, in various workplace settings were considered for inclusion. Studies involving non-allied health professionals (e.g., doctors, nurses), students, or other general community members were excluded during the screening and selection process, as they did not align with the focus on allied health professionals (AHPs). Additionally, studies that exclusively involved psychologists and/or counsellors were excluded. This decision was based on the fact that psychologists and counsellors typically specialize in mental health, which is central to their practice. In contrast, other AHPs often do not have mental health as a core component of their professional training and scope of practice [26]. Furthermore, studies where AHPs worked solely in community or school-based settings were also excluded to maintain the focus on broader workplace contexts, as these environments may involve different dynamics and interventions.

#### Concept.

This review considered all types of suicide prevention training delivered to AHPs, regardless of the mode of delivery (e.g., online, in-person) or the duration of the program. The primary focus was on training that aimed to enhance suicide prevention knowledge, skills, or practices among AHPs.

#### Context.

No limiters were applied during the search regarding country of origin or publication language. This allowed the capture of all relevant studies that outlined suicide prevention training delivered to AHPs as CPD or in-service training.

#### Types of sources.

This scoping review focused solely on empirical studies that met the inclusion criteria. Specifically, only studies that provided suicide prevention training to AHPs and measured its impact, were considered. To maintain the empirical nature of the review, non-empirical sources such as reviews, protocols, policy documents, and opinion pieces were excluded. The rationale for excluding these types of sources is that they often do not provide original data or measurable outcomes regarding the effectiveness of interventions. Policy documents and opinion articles, in particular, may offer valuable insights into broader frameworks or perspectives but do not typically present empirical evidence on the impact of suicide prevention training. Therefore, these were excluded to ensure that the review focused on measurable outcomes directly related to the research questions.

## Materials and methods

This scoping review utilized the methodological framework provided by the Joanna Briggs Institute (JBI) for conducting scoping reviews [27] to assess and synthesize the evidence surrounding suicide prevention training for AHPs within health care environments. This multi-step approach includes identifying the research questions, developing inclusion criteria that align with the study objectives, performing a systematic search, selecting and extracting the evidence, synthesizing and analyzing the evidence and, lastly, summarizing and presenting the findings [27,28]. To facilitate transparency of reporting, this scoping review employed the use of the Preferred Reporting Items for Systematic Reviews and Meta-Analyses extension for Scoping Reviews (PRISMA- ScR) Checklist [29]. Furthermore, in line with PRISMA- ScR guidelines, the protocol for this review was registered with the Open Science Framework (OSF).

### Search strategy

The search strategy for this scoping review was developed in consultation with a health science librarian and aimed to locate both published and unpublished primary studies. In accordance with the JBI framework, a three-step search strategy was adopted [27], including an initial limited search of MEDLINE and Scopus to compile a list of keywords and useful subject headings. This was followed by a second comprehensive search (see S1 Appendix for complete strategy), including all identified key terms and MeSH headings in combination with Boolean operators, with appropriate adaptations made for each database used. Final search and extraction from all databases were conducted on the same day in April 2021. The final search was repeated in July 2023 to capture literature published since April 2021, with the search terms updated to expand the scope of articles found. The authors of papers were contacted where required to request full papers that were not available through the authors’ institutional library access. The databases included in the initial search were MEDLINE (Ovid), CINAHL (EBSCOhost), PSYCHinfo (EBSCOhost) and Scopus. Embase was also included in the updated search. These databases were chosen for their relevance in health-related topics and particularly allied health. Grey literature was also searched using key terms on Trove, OpenGrey and Google Scholar. A final hand search of the included article reference lists was conducted to identify any additional sources.

Studies published from January 2010-July 2023 were included to comprehensively cover applicable literature. The index year of 2010 was chosen as this yields the most recent evidence surrounding current practice for suicide prevention training. This is important due to the increase in suicide rates over the last decade, alongside the changes seen in the experience of suicidality and societal changes, such as the increased use of technology, including online platforms, which have also influenced health care provision [30].

### Study selection

The references retrieved from the systematic search were imported from each database into Endnote X9 (Clarivate Analytics, PA, USA) and duplicates removed. Following the JBI guidelines, the review process was carried out independently by two sets of reviewers at different stages: JR and AR in April 2021, and AG and AR in July 2023. The first stage involved a screening of titles and abstracts by each reviewer to identify potentially relevant papers, followed by full-text retrieval of identified sources for further review against the inclusion criteria.

The final determination of included articles was made through a collaborative review, where the reviewers discussed any discrepancies regarding study eligibility. If there were differences in opinion on whether a study should be included, these were resolved through discussion. The aim was to reach a consensus, ensuring a thorough evaluation of the studies. In cases where consensus could not be reached, a third independent reviewer was available to adjudicate and assist in making a final decision. However, the need for a third reviewer did not arise in this study, as all discrepancies were resolved through dialogue between the primary reviewers.

This multi-step process, which incorporated independent review and collaborative resolution of discrepancies, contributed to the transparency and reliability of the study selection process, ensuring that all relevant studies were consistently identified and included.

### Data extraction

The full-text articles of the final papers selected were retrieved and analyzed using an adaptation of the JBI data extraction instrument [27]. The data extracted from each of the articles included the following study information: Author(s), year of publication, country, target population/study participants, study aim, measures used and outcomes. In addition, the following relevant data pertaining to the research questions were included: type of training, training mode of delivery, duration of training, underpinning framework/practice model, measurement tools used and outcomes. These domains were chosen to facilitate the mapping of key concepts and comprehensive evaluation of the relevant literature, in accordance with the aims of this scoping review.

### Data analysis and presentation

Public health related research and interventions are typically complex and multi-faceted, making synthesis and integration of findings challenging [31]. As this review considered mixed research methodologies, the extracted data was collated and presented in tabular form to facilitate analysis. Individual studies were summarized under the different domains of the table, enabling common themes to be categorized in alignment with the review questions and differences or contradictions noted. A content synthesis was conducted to accompany the summary table. This involved defining categories of focus, comparison and pooling of data, and providing a descriptive account of the characteristics of the findings. This is useful for determining causal relationships and generalizability of results from a range of studies [32].

## Results

### Study inclusion

A total of 1665 articles were retrieved in April 2021. Following the removal of 612 duplicates, 1053 records were screened by title, 149 records were screened by title and abstract, and 80 articles were reviewed for eligibility. The search in July 2023 yielded 3032 results. After 571 duplicates were removed, 2461 articles were screened by title, 95 articles were screened by title and abstract, and 26 articles were reviewed for eligibility. The grey literature search yielded multiple duplicate results but did not provide any additional relevant articles.

**Fig 1:**
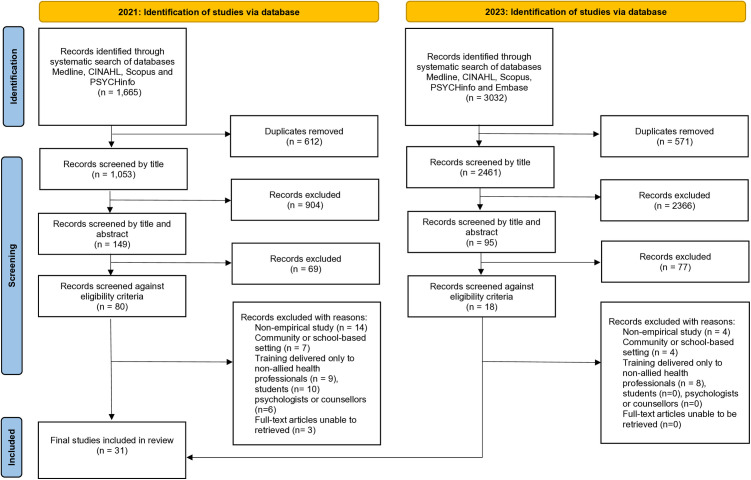
PRISMA flowchart of search strategy and study selection.

Forty-nine articles were excluded in April 2021 and 16 articles were excluded in July 2023 for not meeting the eligibility criteria. Reasons for exclusion included: study did not provide empirical data, discussed prior training, or gave descriptions of training programs without delivery (n = 18); participants were students (n = 10); participants were non-allied health professionals (n = 17); participants were only psychologists or counsellors (n = 6); study setting was school- or community-based (n = 11); full-text unable to be retrieved (n = 3) (see S2 Appendix for a full list of excluded articles). A PRISMA Flowchart of the search strategy and study selection is presented in Fig 1.

A complete summary of all included papers can be found in S3 Appendix, which includes thirty two peer-reviewed journal articles and one published doctoral thesis. Table 1 provides an abbreviated summary of the included studies and suicide prevention training characteristics.

**Table 1:** Summary of study and training characteristics.

Year	Author(s)	Country	Study Design	Study population	Sample size	Type of training	Delivery mode	Duration
2018	Adrian et al.	US	Case Series	Mixed cohort (P, SW, MH, O)	75	Skills-based	Face-to-face	6-hour workshop
2019	Condron et al.	US	Quasi-experimental	Mixed cohort (MH, SW, O)	1,918	Gatekeeper – compared two types	Face-to-face	1–2-hour workshop vs 2-day workshops
2019	Cramer et al.	US	Case Series	Mixed cohort (N, P, M, SW, C)	43	Competency-based	Online	20 hours (30-day completion)
2011	da Silva Cais et al.	Brazil	Case Series	Mixed cohort (M, P, OT, SW, N)	135	Skills-based	Face-to-face	18 hours (2-day) workshop
2013	Donald et al.	AUS	Case Series	Mixed cohort (N, M, P, SW, OT, O)	242 (55)	Non-specific – compared two types	Face-to-face	1-day workshop vs 3-day workshops
2017	Duvivier, L.	US	RCT	Mixed cohort (P, SW, MH, M, O)	163	Skills-based	Online	2-week completion
2019	Gask et al.	Scotland	Qualitative	Mixed cohort (N, P, OT, M, O)	60	Skills-based	Face-to-face	2-4-day workshops
2012	Gomez et al.	Chile	Case Series	Mixed cohort (P, OT, SW, PT, N, M)	89	Skills-based	Face-to-face	2-day workshops
2017	Gryglewicz et al.	US	Case Series	Mixed cohort (N/S)	178	Gatekeeper	Online	8-12 hours (30-day completion)
2020	Gryglewicz et al.	US	Case Series	Mixed cohort (N/S)	178	Role-play	Face-to-face	4.5-hour workshop
2022	Hawgood et al.	AUS	Case Series	Mixed cohort (N/S)	222	Competency-based	Face-to-face	2-day workshops
2012	Huh et al.	US	Case Series	Mixed cohort (SW, P, N, M, OT, O)	67	Skills-based	Face-to-face	6.5-hour workshop
2012	Jacobson et al.	US	Case Series	Mixed cohort (C, SW, P, N, M)	194	Skills-based	Online & Face-to-face	2-day workshop (unknown time for online modules)
2011	Johnson et al.	US	Case Series	Mixed cohort (N/S)	111	Means restriction	Face-to-face	2-hour workshop
2018	Jones et al.	AUS	Qualitative	Mixed cohort (C, SW, O)	24	Non-specific	Face-to-face	1-day workshop
2020	Kawashima et al.	Japan	Case Series	Mixed cohort (M, N, SW, P, O)	274	Assertive case-management	Face-to-face	2-day workshops
2019	La Guardia et al.	US	Case Series	Mixed cohort (C, P, SW, N, O)	29	Competency-based	Face-to-face	Half-day workshop
2011	Levitt et al.	US	Case Series	Social workers	120	Gatekeeper	Face-to-face	Half-day workshop
2014	Matthieu & Swensen.	US	Case Series	Mixed cohort (SW, C, O)	39	Gatekeeper	Face-to-face	1-2 hours
2020	Midorikawa et al.	Japan	Case Series	Mixed cohort (M, N, SW, O)	115	Gatekeeper	Face-to-face	Half-day workshop
2016	Mirick et al.	US	Case Series	Mixed cohort (SW, MH, C, P, N, OT, O)	442	Competency-based	Face-to-face	6-hour workshop
2020	Mirick et al.	US	Qualitative	Mixed cohort (SW, MH, P, O)	60	Competency-based	Face-to-face	6-hour workshop
2018	Painter et al.	US	Case Series	Pharmacists	77	Gatekeeper	Face-to-face	1-2-hour workshop
2012	Pisani et al.	US	Case Series	Mixed cohort (C, P, M, N, SW, O)	338	Competency-based	Face-to-face	3-hour workshop
2016	Pullen, J.	US	Case Series	Mixed cohort (N, SW, PT, OT, O)	43	Gatekeeper	Face-to-face	2-hour workshop
2021	Scott, M.	US	Mixed methods	Social workers	22	Skills-based	Face-to-face	2-day workshops
2016	Serra-Taylor et al.	Puerto Rico	Case Series	Mixed cohort (C, P, SW)	39	Competency-based	Face-to-face	8-hour workshop
2010	Shim et al.	US	Case Series	Mixed cohort (N, SW, O)	54	Non-specific	Face-to-face	3-hour workshop
2019	Slovak et al.	US	Mixed Method	Mixed cohort (N, SW, O)	60	Means restriction	Face-to-face	5-hour workshop
2019	Stallman, H.	AUS	Case Series	Mixed cohort (P, N, SW, C, OT, M)	302	Needs- and Strengths-based	Online	8 hours
2023	Stuber et al.	US	Case Series	Mixed cohort (N/S)	1548	Skills-based	Online	6 hours
2018	Terpstra et al.	Netherlands	Case Series	Mixed cohort (N/S)	174	Gatekeeper	Face-to-face	4-hour workshop
2014	Wharff et al.	US	Quasi-experimental	Social workers	72	Competency-based	Face-to-face	2x 1.5-hour workshops

P = Psychology, N = Nursing, SW = Social Work, C = Counselling, OT = Occupational Therapy, PT = Physical Therapy, M = Medicine, MH = Mental Health, Ph = Pharmacy, O = Other, N/S = Not specified.

### Characteristics of included studies

Among the included studies there were twenty-five case series, two quasi-experimental studies, one randomized controlled trial (RCT), three qualitative studies, and two that combined a case series and a qualitative approach (i.e., mixed methods). The majority of studies were conducted in the United States (n = 22), with the remaining studies heralding from Australia (n = 4), Japan (n = 2), and one each from Scotland, Netherlands, Brazil, Chile, and Puerto Rico (see Table 1 for details).

The sample sizes ranged from 22 to 1918 participants, with ten of the studies recording between 100–200 participants. Twelve studies reported a 10–30% drop-out rate and nine studies an approximate 50–70% drop-out, whereby participants completed the training but not the study evaluations. Four studies reported a 100% response rate for all intervention participants [33–36], and three studies between 95–100% response rate [37–39]; however, their intervention participation ranged between 34–71%. One study did not specifically report an intervention participation or response rate [40]. The three qualitative studies chose a manageable number of participants (n = 24–60) from a larger pool of training participants (n = 248–1155) [41–43].

Four studies provided no demographic data for the participants. Of the studies that reported on gender, the prevalence of high female participation was considerable, with 27 of the 29 reporting a female majority. Twenty-one studies provided data on age, with the reported mean age of participants ranging from 31.7 to 48.3 years.

The study designs and procedures were greatly varied. Most of the studies used questionnaires for their measures (n = 29), though some also used vignettes and/or documentation exercises (n = 3), and one included a focus group. The majority of case series studies had pre and post measures delivered immediately after training (n = 19), some added a follow-up measure that was typically administered 3 months post-training (n = 5), and one did not administer a baseline measure, only post-training and follow-up. The quasi-experimental studies delivered follow-up measures only, at 3 months post-training (n = 2). The RCT adopted a pre-post design (immediate), while the mixed method studies differed, as one had pre, post and follow-up (3 months) and the other post and follow-up measures only (3 and 5 months). The three qualitative studies were follow-up only, undertaking telephone interviews either at 3 months (n = 1) or 1–2 years (n = 2) post-training.

### Review findings

#### Types of suicide prevention training.

The types of training reported in the studies varied significantly. Skill-based training (n = 9) was most widely used, followed by gatekeeper training (n = 8) and competency-based training (n = 8). Other forms of training included means restriction (n = 2), role-play, needs- and strengths-based and assertive case management (n = 1 each).

The predominant mode of delivery for the training programs was face-to-face workshops (n = 27), which commonly comprised of lectures, video presentations, group discussions, role-rehearsal, case study analysis and printed resources. The duration of the training programs was also widely varied, ranging from 1 to 20 hours. The most common duration of the face-to-face workshops was 4–6 hours (n = 8) and 2–3 days (n = 8). Studies with online delivery varied from 6–20 hours of content, with 1–4 weeks available for participants to complete all modules.

#### Allied health disciplines targeted for training.

Most of the included studies reported a mixed cohort of participants (n = 29), while the remaining were designed for participation by single disciplines including social workers (n = 3) and pharmacists (n = 1). The mixed cohorts were diverse; however, five did not report on the specific participant professions, and one only partially described specific participant professions included. The predominant allied health professions of interest represented in the mixed cohort studies were social workers (n = 21) and occupational therapists (n = 8). The number of mixed cohort studies specifically targeting AHPs only was small (n = 6), as most of the studies were aimed at health professionals more generally and commonly included nursing and other medical professions.

#### Underpinning frameworks and constructs.

The frameworks, models, tools, and approaches adopted by the included studies were varied (Table 2). These were grouped into the following seven categories and constructs: (i) Knowledge – epidemiology, risk and/or protective factors, intervention, lethal means and cognitive behavioral therapy (CBT); (ii) Skills – risk assessment, identification, psychoeducation, crisis management, intervention and postvention; (iii) Behavior – attitudes, responses and clinical practice; (iv) Cultural considerations – epidemiology, risk factors and context; (v) Standard of care – therapeutic alliance, engagement, documentation and liability; (vi) Reflective discussion – open group discussions, participant questions and concerns; (vii) Self-care – debriefing and workplace safety.

**Table 2:** Summary of training frameworks and constructs.

Publication	Sample size	Type of training	Theoretical Framework and/or Tools	Key Constructs
Adrian et al. (2018)	75	Skills-based	Non-specific *Suicide Risk Decision Tool (SRDT)*	K, S, B, C
Condron et al. (2019)	1,918	Gatekeeper – compared two	Question, Persuade, Refer (QPR) Applied Suicide Intervention Skills Training (ASIST)	K, S, C, SC
Cramer et al. (2019)	43	Competency-based	Non-specific	K, B, SoC, S
da Silva Cais et al. (2011)	135	Skills-based	Non-specific	K, S, B
Donald et al. (2013)	242 (55)	Non-specific – compared two	Non-specific	K, S, B, C
Duvivier. L. (2017)	163	Skills-based	Decision Tree Model of Suicide Risk Assessment	K, S, B
Gask et al. (2019)	60	Skills-based	Skills Training On Risk Management (STORM) Model	K, S, R
Gomez et al. (2012)	89	Skills-based	Non-specific	K, S, SoC
Gryglewicz et al. (2017)	178	Gatekeeper	Question, Persuade, Refer, Treat (QPRT) *QPRT Suicide Risk Management Inventory*	K, S, SoC
Gryglewicz et al. (2020)	178	Role-play	Question, Persuade, Refer, Treat (QPRT)	K, S, SoC, B
Hawgood et al. (2022)	222	Competency-based	Systematic Tailored Assessment for Responding to Suicidality (STARS)-2	K, S, B, SoC, SC
Huh et al. (2012)	67	Skills-based	Non-specific	K, B, SoC
Jacobson et al. (2012)	194	Skills-based	Recognizing and Responding to Suicide Risk (RRSR) *CASE approach*	K, S, B
Johnson et al. (2011)	111	Means restriction	Counselling on Access to Lethal Means (CALM)	K, S, SoC
Jones et al. (2018)	24	Non-specific	Non-specific – tailored for regional context	K, C, SoC, R, SC
Kawashima et al. (2020)	274	Assertive case-management	Assertive case-management training	K, S, SoC, R
La Guardia et al. (2019)	29	Competency-based	Zero Suicide Model	K, S, B, SoC
Levitt et al. 2011	120	Gatekeeper	Non-specific	K, SoC, S
Matthieu et al. (2014)	39	Gatekeeper	Question, Persuade, Refer (QPR)	K, SoC, S
Midorikawa et al. (2020)	115	Gatekeeper	Non-specific *TALK steps (Tell, Ask, Listen and KeepSafe)*	K, B
Mirick et al. (2016)	442	Competency-based	Suicide Assessment and Intervention Training (SAIT)	K, SoC, S, B, SC
Mirick et al. (2020)	60	Competency-based	Suicide Assessment and Intervention Training (SAIT)	K, SoC, S, B, SC
Painter et al. (2018)	77	Gatekeeper	Non-specific – based on QPR, tailored for pharmacists	K, SoC, S, B, SC
Pisani et al. (2012)	338	Competency-based	Commitment to Living: Understanding and Responding to Suicide Risk (CTL)	K, S, B
Pullen, J. (2016)	43	Gatekeeper	Question, Persuade, Refer (QPR)	K, SoC, S
Scott, M. (2021)	22	Skills-based	Cognitive Behavioural Therapy for Suicide Prevention (CBT-SP)	K, SoC, S
Serra-Taylor et al. (2016)	39	Competency-based	Non-specific	K, SoC, S
Shim et al. (2010)	54	Non-specific	Managing Suicidality in the Emergency Department *The Basic Suicide Assessment Five-step Evaluation (B-SAFE) Protocol*	K, SoC, S
Slovak et al. (2019)	60	Means restriction	Counselling on Access to Lethal Means (CALM)	K, SoC, S, R
Stallman, H. (2019)	302	Needs- and Strengths-based	Care.Collaborate.Connect: Suicide Prevention training	K, SoC, S, SC
Stuber et al. (2023)	1548	Skills-based.	Non-specific	K, S, B
Terpstra et al. (2018)	174	Gatekeeper	Non-specific – based on QPR, tailored for application in the Netherlands	K, SoC
Wharff et al. (2014)	72	Competency-based	Non-specific *Suicide Assessment Five-Step Evaluation and Triage (SAFE-T) procedure*	K, S, SoC

K= Knowledge, S= Skills, B= Behaviour, C= Cultural considerations, SoC= Standard of Care, R= Reflective discussion, SC= Self-care.

#### Skills-based training.

The skills-based training programs included content to enhance suicide prevention knowledge; however, there was a stronger focus on risk identification and assessment, crisis management and psychoeducation. This type of training also commonly had components designed to influence the behaviors and/or attitudes of participants towards suicide. Four of the six that had face-to-face workshops were delivered across 2–3 days [33,39,41,44], while the other two were 6-hour programs [45,46]. Two training programs were delivered online, one allowing 2 weeks for completion [47], with the other not describing the time provided for completion [48]. One training program had blended online delivery with a 2-day face-to-face workshop [49].

Of the eight studies that used skills-based training, five were non-specific (i.e., designed specifically for the study) [33,44–46,48], while the remaining studies each utilized a different approach. These included the Decision Tree model [47], Cognitive Behavioral Therapy for Suicide Prevention (CBT-SP) [39], Recognizing and Responding to Suicide Risk (RRSR) [49] and the Skills Training on Risk Management (STORM) model [41]. Additionally, one study taught participants how to use the Suicide Risk Decision Tool (SRDT) [45] and another the CASE Approach [49] to support their clinical suicide risk assessments.

#### Gatekeeper training.

The gatekeeper programs typically aimed to increase knowledge and skills in identification, assessment, and management of suicidal behaviors; cultural context and risk factors; and therapeutic alliance. Question, Persuade, Refer (QPR) and/or content based on QPR was the most widely used gatekeeper program (n = 6), which is typically delivered in 1–2-hour workshops. One study compared QPR with Applied Suicide Intervention Skills Training (ASIST), a different gatekeeper program delivered across a 2-day workshop [37]. Two other non-specific gatekeeper training programs were reported that were specifically designed for the studies [50,51]. One study also exposed participants to the QPRT Suicide Risk Management Inventory used in clinical practice to aid suicide management [52].

#### Competency-based training.

The competency-based programs targeted learning around the core competencies of suicide risk assessment and management, including understanding suicide, risk/protective factors, therapeutic alliance, management of responses to client, formulation of risk, crisis intervention and safety planning. Seven of the eight programs were delivered in face-to-face workshops ranging from 3 hours to two days in length [34,43,53–57]. Cramer et al. [58] opted for online delivery, allowing participants 30 days to complete 20 hours of content.

Four of the programs were non-specific, using prior studies and/or professional guidelines to design training content [34,53,57,58]. Both studies conducted by Mirick et al. [43,55] delivered the Suicide Assessment and Intervention Training (SAIT) program, whereas Pisani et al. [56] provided Commitment to Living (CTL) training, and La Guardia et al. [54] used the Zero Suicide Model multi-level framework. Wharff et al.’s [57] was the only study from this category to teach participants the use of an additional tool called the Suicide Assessment Five-Step Evaluation and Triage (SAFE-T) procedure, often utilized in hospital settings to quickly assess level of suicide risk.

#### Other types of training.

The means-restriction program Counselling on Access to Lethal Means (CALM) was adopted by two studies, delivering content in brief face-to-face workshops that aim to increase knowledge of epidemiology and risk factors pertaining to lethal means; skills in identification, assessment, and intervention; and a strong focus on therapeutic alliance and client engagement [59,60]. One study utilized an assertive case management approach, which addressed psychoeducation, client engagement and intervention skills across a 2-day workshop [61]. Another study adopted the needs- and strengths-based framework Care.Collaborate.Connect, comprising 8 online modules emphasizing therapeutic alliance, positive attitudes and responses to suicidal behavior, and psychoeducation [36].

Three studies did not utilize any specific frameworks, but instead tailored the training for the context or targeted participants. One was designed for face-to-face delivery (1-day) to participants that worked in remote communities, with many engaging with indigenous populations [42]. This covered suicide prevention content specific to the region, including a lived experience component, and aimed to increase participants’ confidence in addressing suicidality in their practice. Another was a 3-hour workshop designed for emergency department personnel and included exposure to the Basic Suicide Assessment Five-step Evaluation (B-SAFE) Protocol, a tool administered to assess immediate risk and provide brief intervention strategies [35]. The third compared brief and enhanced training programs (1-day versus 3-day workshops), that delivered content regarding suicide crisis identification and intervention [50].

#### Reported effectiveness of training.

A diverse range of measurement tools were used in the studies (see Table 3 for a summary), with many using more than one measurement tool (n = 17) and/or a combination of developed and study-specific measures (n = 9). Twenty-seven studies developed their own tools, which included questionnaires, documentation exercises and interview questions. Fifteen studies administered previously developed tools, including 24 validated measures and 3 not validated. Of the validated tools, only 5 were used in more than one study: Suicide Behavior Attitude Questionnaire (SBAQ) [33,44,47,54,58], Suicide Competency Assessment Form (SCAF) [54,58], Attitudes Towards Suicide (ATTS) [51,61], Attitudes toward Self-Harm Patients (ASHP) [54,58] and Suicide Interventionist Response Inventory (SIRI-2) [45,61]. Many of the developed tools were adapted and/or only a portion of the tool was used (e.g., one subscale) to suit the needs of the study.

**Table 3:** Summary of measures and results.

Publication	Study Design	Type of training	Measures Used	Measures Taken	Results
Adrian et al. (2018)	Case Series	Skills-based	• Suicide Interventionist Response Inventory (SIRI-2) • Attitudes Toward Standard Assessment Scales-Adapted (ASA) • Suicide Intervention Questionnaire (SIQ)	Pre; Post; Follow-up (3 months)	• Significant increases were found in suicide assessment knowledge (p = 0.02) and attitudes towards intervention (p = 0.01); both were maintained at T3 • Self-rated suicide risk knowledge and applied knowledge did not change significantly from T1 – T2 or T3 (p = 0.22) • Training evaluated by participants as highly relevant and effective • 52% plan to change their treatment approach, 89% to change their communication
Condron et al. (2019)	Quasi-experimental	Gatekeeper – compared two types	• Training Utilization and Preservation Survey (TUP-S)	Follow-up (3 months)	• Improved performance for in-depth training (ASIST) compared with brief (QPR) training across 3 main domains (i) Identifying risk (p = 0.06), (ii) referring to general services (p = 0.001) and (iii) referring to acute services (p = 0.03). • The results suggest that in-depth gatekeeper trainings modify behavior more than brief trainings
Cramer et al. (2019)	Case Series	Competency-based	• Need for Affect Questionnaire-Short Form (NAQ-S) • The Suicide Behavior Attitude Questionnaire (SBAQ) • Attitudes toward Self-Harm Patients Scale (ASHP) • Suicide Competency Assessment Form (SCAF) • Study specific – 15-question multiple-choice knowledge quiz	Pre; Post	• Large gains in SP knowledge & perceived skill ability, capacity to work with suicidal clients and ability to assess & manage risk • Moderate gain for perceived ability to help self-harming patients • Small-to-moderate decrease in negative attitudes towards self-harm
da Silva Cais et al. (2011)	Case Series	Skills-based	• Suicide Behavior Attitude Questionnaire (SBAQ) • Suicide Prevention Knowledge Questionnaire (SPKQ)	Pre; Post	• Statistically significant increases in SP knowledge (p < 0.001); and attitudes across domains (i) suicide right (p = 0.02), (ii) negative attitudes (p = 0.002) and (iii) professional capacity (p < 0.001) • The training led to improvement in knowledge and attitudes for all ages and occupations despite prior knowledge
Donald et al. (2013)	Case Series	Non-specific – compared two types	• Study specific – a 25 item questionnaire evaluating: (1) recognizing the signs of suicide, (2) crises intervention strategies (3) brief counselling techniques (4) postvention strategies (5) expansion of organizational networks	Pre; Post; Follow-up (3 months)	• Standard training produced increase in knowledge at T2 & T3; however, enhanced training produced statistically significant increase in knowledge (p = 0.001) • Enhanced training produced significant increases in expansion of organisation networks across domains (i) information exchange (p < 0.001), (ii) liaison & support (p = 0.01) and (iii) local planning (p = 0.001) • Findings support the use of enhanced training for improved SP knowledge and changes to clinical practice
Duvivier. L. (2017)	RCT	Skills-based	• Computer-Based Training Attitudes Scale (CBTAS) • Motivated Strategies for Learning Questionnaire (MSLQ) • Study specific – 64 item questionnaire evaluating: (1)Knowledge – 20 items (2)Skill – 36 items (3)Training satisfaction – 8 items	Pre; Post	• Group 1 (training) scored significantly higher than Group 2 (control) across all domains (knowledge, skill, risk assessment, rating risk factors, self-efficacy) • No significant difference found in ‘attitudes’ between the groups • Prior SP training did not moderate learning effect • Overall, participants were satisfied with the content & delivery of the training
Gask et al. (2019)	Qualitative	Skills-based	• Semi-structured telephone interviews	Follow-up (1–2 years)	• Facilitators & participants noted positive effect on clinical practice post-training • Training improved participants confidence in asking patients about suicidal behaviours and their ability to identify risks • Facilitators gained confidence and reassurance from working in pairs and seeking supervision to manage problems • Facilitators noted that implementing further training is difficult when not mandated
Gomez et al. (2012)	Case Series	Skills-based	• Suicide Behavior Attitudes Questionnaire (SBAQ) • Attitudinal Beliefs Questionnaire about Suicidal Behavior (CCCS-18) • Study-specific instrument based on clinical vignettes	Pre; Post	• Significant changes were evidenced on 3 SBAQ subscales (all p < 0.003) • Significant changes were obtained four subscales of the CCCS-18 (all p < 0.001) • Positive attitudinal changes and a decrease of negative feelings toward the patient and an increase in the perception of professional competence
Gryglewicz et al. (2017)	Case Series	Gatekeeper	• Study specific – 45 item questionnaire evaluating: (1) Knowledge – 17 items (2) Attitudes – 5 items (3) Social norms – 6 items (4) Perceived behavioural control (PBC) – 3 items (5) Cultural competence – 6 items (6) Training satisfaction – 6 items	Pre; Post	• Significant increase in total knowledge; risk judgement, crisis management; PBC; confidence and risk/protective factors (p < 0.001) • No significant changes in knowledge of legal issues or safety planning • Overall satisfaction with training and perceived ability to integrate knowledge into practice • 77% of participants rated training as culturally competent
Gryglewicz et al. (2020)	Case Series	Role-play	• Study specific – 28 item questionnaire evaluating: (1) Attitudes – 4 items (2) Subjective norms – 6 items (4) Perceived behavioural control (PBC) – 18 items • Study specific – 15 item survey evaluating (1) Training satisfaction (2) Trainer competence (3) Alliance with trainer (4) Training utility	Pre; Post	• There was a 40% increase in subjective norms and 67% increase in PBC • There was no significant increase in perceived confidence. • 97% of participants felt the trainers were attentive and 85% were comfortable participating in role-play. • Almost 94% of participants reported their rapport/trust of the trainer helped reduce any role-play anxiety/fear. • 99% reported the training as valuable and skill enhancing.
Hawgood et al. (2022)	Case Series	Competency-based	• Attitudes to Suicide Prevention Scale (ASP) • Perceived Capability Scale • Declarative Knowledge Scale • Reluctance to Intervene Scale	Pre; Post	• Statistically significant improvements in attitudes to suicide prevention(p = 0.014), declarative knowledge (p < 0.001) and perceived capability (p < 0.001) were found. • Reluctance to intervene did not significantly improve as a result of training (p = 0.123).
Huh et al. (2012)	Case Series	Skills-based	• Study specific – survey evaluating: (1)Knowledge (2)Attitudes (3)Confidence • Study specific – Clinical behaviour assessment: Case notes reflecting assessment & management plan based on vignette	Pre; Post; Follow-up (3 months)	• Increases in general suicide risk assessment and management skills and perceived confidence (sustained at T3) • Significant improvement in all participants’ overall medical record documentation quality and conceptual clarity regarding static versus dynamic risk • 43% of participants reported incorporating skills/knowledge into clinical practice at T3 • 90% of participants maintained increased awareness and 85% would like to learn more about late-life suicide at T3
Jacobson et al. (2012)	Case Series	Skills-based	• Attitudes to Suicide Prevention scale (ASP) • Counselling Self-Estimate Inventory (COSE) • Suicide Behavior Attitude Questionnaire (SBAQ) • Study specific – Clinical Risk Management Scale: 9 items measured self-confidence in management of clinical risk • Modified STORM survey to assess: (1)Confidence to work with at risk clients – 3 items (2)Frequency of skill use – 3 items (3)Practice behaviours related to suicide intent – 9 items	Pre; Post; Follow-up (4 months)	• Statistically significant results for mean differences in SBAQ scores; ‘Clinical Risk Management scale’ and confidence ratings for the modified STORM (p < .001) across time and maintained (T3) • ‘Assessment of Suicide Intent’ scores were statistically significant (p = 0.02) • Statistically significant increase in perceived effectiveness of suicide safety plans (p = 0.01) and vignette analysis (p = 0.02) • 91% of participants reported implementation of skills at T3
Johnson et al. (2011)	Case Series	Means restriction	• Study specific – 11 item questionnaire evaluated: (1)Knowledge (2)Attitudes (3)Beliefs (4)Behavioural intentions about counselling on lethal means	Post; Follow-up (6–8 weeks)	• Majority of participants agreed that CALM training offered useful strategies; met an important need and that they would implement the skills learned (>80%) • At T3, 65% of participants reported skill implementation post-workshop • Limitations include: no baseline data, lack of objective data, attention bias and lack of evaluation of provider by client pre/post training
Jones et al. (2018)	Qualitative	Non-specific	• Study specific – interview questions (open-ended) to help understand the impact and application of the training in clinical practice	Follow-up (~3 months)	• Participants placed high value/expressed strong appreciation of the lived experience component of the training, stating this reduced suicide related stigma • The training empowered them to work with at risk clients (increased confidence) • Participants reported implementing the training skills • Participants reported the importance of location/regional area specific training • Suggestions were made to increase training duration and offer training annually (to capture new employees)
Kawashima et al. (2020)	Case Series	Assertive case-management	Japanese versions of: • Attitudes to Suicide Prevention Scale (ASP) • Gatekeeper Self-Efficacy Scale (GKSES) • Suicide Intervention Response Inventory (SIRI) • Attitudes Toward Suicide Questionnaire (ATTS) • Study specific – training satisfaction measure	Pre; Post	• Increased self-efficacy, favourable attitudes to suicide prevention and suicide intervention skills with significant increases in Attitudes to Suicide Prevention Scale; Gatekeeper Self-Efficacy Scale; SIRI-1 and SIRI-2 (all p < 0.001) • Effect of training was highest in those who had no prior suicide prevention training • The training had a higher impact on males than females, which was consistent with SIRI scores from previous studies
La Guardia et al. (2019)	Case Series	Competency-based	• Study specific – Suicide Risk Assessment and Management Knowledge – 12 item multiple-choice quiz based on training content • Suicide Competency Assessment Form (SCAF) • Suicide Behavior Attitude Questionnaire (SBAQ) • Attitudes toward Self-Harm Patients (ASHP) • Interprofessional Socialization and Valuing Scale (ISVS) (T1 only)	Pre; Post	• Statistically significant results found for – Knowledge (p < 0.001); Competency (p = 0.001); Perceived ability (p < 0.001); Improved Optimism and Patience (p = 0.04); Confidence and Adequacy of training (p = 0.008) and Professional Capacity (p < 0.001) • ‘Negative feelings towards patients’ was improved but not significantly (p = 0.71) • Evidence of a connection between interprofessional socialization and perceived self-efficacy of working with suicidal individuals
Levitt et al. (2011)	Case Series	Gatekeeper	• Study specific – 23 item multiple choice test evaluated epidemiology and prevention, with roughly half the test items addressing each area (specific domains not provided).	Pre; Post; Follow-up (21 months)	• Statistically significant gains made across all domains at T2 (p < 0.001) • T3 scores were slightly lower than T2, but remained higher than T1 • Training was effective in increasing the suicidality-related knowledge as demonstrated by both subjective and objective measures, and this benefit was sustained
Matthieu et al. (2014)	Case Series	Gatekeeper	• Study specific – 42 item questionnaire evaluated: (1)Perceived self-efficacy −10 items (2)Declarative knowledge −14 items (3)Satisfaction and impact of training – 4 items (4)Awareness of resources and referrals – 4 items (5)Behavioural rehearsal session was evaluated using −10-items	Pre; Post	• 80% of participants reported increased awareness of the risk factors for suicide. • Self-efficacy scores showed a statistically significant increase (p = 0.00) • Participants rated training satisfaction as “high” • 1/5 of participants reported knowledge of SP efforts in their workplace • Only a small number of participants (<25%) were aware of available external resources
Midorikawa et al. (2020)	Case Series	Gatekeeper	Japanese versions of: • General Health Questionnaire (GHQ-12) • Attitudes Towards Suicide (ATTS)	Pre; Post	• 23.5% of participants were classified as having poor mental health • A significant difference in attitude towards suicide between the 2 groups (good vs poor mental health) • Gatekeepers with poorer mental health may be negatively biased toward counter-suicide intervention
Mirick et al. (2016)	Case Series	Competency-based	• Study specific – 25 item questionnaire evaluated: (1)Knowledge (2)Confidence (3)Prior exposure to SP training	Pre; Post	• All participants demonstrated increased knowledge and confidence regardless of prior training • Differences in training affect noted between those with prior training compared to those without • Participants reported safety planning and assessment tools as valuable training components
Mirick et al. (2020)	Qualitative	Competency-based	• Study specific – semi-structured interview questions that explored changes to clinical practice post SP training	Follow-up (1–2years)	• 90% reported changes to practice including (i) approach to conversations; (ii) management of clients; (iii) stronger relationships. • Findings suggest the training was incorporated into practice beyond changed knowledge and attitude and that exposure to current best-practices may be enough for participants to adjust their own practices
Painter et al. (2018)	Case Series	Gatekeeper	• Study specific – 25 item questionnaire evaluated: (1)General perception (2)Self-efficacy (3)Attitudes	Pre; Post	• There was no statistically significant difference in respondents’ general perception of SP • More than 75% of participants reported increases in confidence, self-efficacy and use of appropriate interventions • Statistically significant changes in attitudes toward suicide prevention were observed (p < 0.0001)
Pisani et al. (2012)	Case Series	Competency-based	• Study specific – 22 item questionnaire evaluated: (1)Provider confidence – 9 items (2)Knowledge – 13 items • Documentation of Risk Formulation – case study analysis (clinical vignettes) • Perception of Potential Transfer of Training (PTT) (Post Only Self-Report) – 18 items	Pre; Post	• Participants’ knowledge and confidence scores improved significantly (p < 0.001) • Documentation samples demonstrated significant improvements of core risk formulation skills (p < 0.001) • Participants perceived they had sufficient knowledge, motivation, and resources to implement the educational content in their workplace.
Pullen, J. (2016)	Case Series	Gatekeeper	• Study specific – 9 item survey evaluating: (1)Knowledge (2)Skills (3)Perceptions • Attitudes Toward Suicide Prevention Scale (ATSPS)	Pre; Post	• Increased knowledge and awareness of risk factors; increased risk identification skills • Participants reported increased confidence in discussing suicidality • Participants also reported that they now felt identifying and managing those at risk for suicide was within their domain of professional responsibility and increased comfort in therapeutic communication
Scott, M. (2021)	Mixed methods – Case series and Qualitative	Skills-based	• Study specific – 26 item questionnaire evaluating: (1)CBT (general) – 12 items (2)CBT for SP (techniques) – 14 items • Barrier to Implementation of Evidence-Based Practice Scale – 15 items • Follow-up phone interview – assessed self-reported integration of skills	Pre; Post; Follow-up (3 months)	• Significant increase in knowledge of CBT (general) and CBT-SP skills and knowledge (all p < 0.000). • Prior exposure to CBT did not affect knowledge gains from training • Significant gains were seen for skills in safety planning and understanding CBT • All participants reported learning new techniques and the majority (95%) reported integrating them into their practice
Serra-Taylor et al. (2016)	Case Series	Competency-based	• Study specific – 20 item survey evaluating: (1)Intervention skills (2)Knowledge	Pre; Post	• Statistically significant differences were found between pre and post-tests in 5 constructs relating to knowledge and skills (all p = .000) • There was a 28.7% increase in knowledge and 12.3% increase in skills pre-post, with an overall increase of 20.5% across all survey questions. • Results suggest that an evidence-based training that follows the core competencies and includes practical exercises, increases the knowledge and intervention skills of mental health professionals in the assessment and management of suicide risk.
Shim et al. (2010)	Case Series	Non-specific	• Study specific – 20 item survey evaluating: (1)Knowledge – 16 items (2)Self-efficacy – 12 items (3)Training satisfaction – 4 items	Pre; Post	• Statistically significant increases in knowledge and self-efficacy regarding management of suicidality in the emergency department pre-post (p < 0.001) • 89.5% rated the training as very or extremely helpful and 84.2% as very or extremely relevant • 86.9% reported being “very likely” to use information from the training in their work; and would recommend the training to others
Slovak et al. (2019)	Mixed Methods – Case series and Qualitative	Means restriction	• Study specific – survey evaluating (Post & 3 months) (1)Knowledge (2)Attitudes (3)Intention (4)Demographics • Focus group – open discussion about training (5 months)	Post; Follow-up (3 & 5 months)	• 25% of participants reported having never receiving any formal suicide prevention training, and 92% reported never receiving training in counselling about firearms • 70–80% of participants reported that training was useful in providing concrete knowledge and skills, and was relevant to their work • many of post-test results showed a statistically significant increase at follow-up (5 items were p < 0.05) • Focus group reported that the training increased their knowledge about firearm access, signs and symptoms of suicidality and acquisition of skills and strategies
Stallman, H. (2019)	Case Series	Needs- and Strengths-based	• The Suicide Prevention Training Evaluation Tool (SPTET)	Pre; Post	• Participants showed significant improvements in knowledge, attitudes, confidence, and self-care pre-post (all p < 0.001) • Participants reported online training as useful to their learning and many found it fitted well with their organisations’ values and priorities
Stuber et al. (2023)	Case Series	Skills-based	• Study specific – 21 item questionnaire evaluating: (1)Knowledge (2)Attidudes (3)Confidence	Pre; Post	• Statistically significant improvements in all 4 knowledge questions and all 8 questions measuring confidence in applying suicide prevention skills (p < 0.01). • Attitudes improved in 8 out of 9 questions (p < 0.01), with the greatest improvements in attitudes to conversing about firearms and medication storage.
Terpstra et al. (2018)	Case Series	Gatekeeper	• Study specific – 10 item questionnaire evaluating: (1)Knowledge (2)Confidence (3)demographics	Pre; Post (6 weeks)	• Statistically significant increases in professionals’ knowledge and confidence (p < 0.001) • Prior training exposure did not affect knowledge gains
Wharff et al. (2014)	Quasi-experimental	Competency-based	• Study specific – 25 item questionnaire evaluating: (1)Understanding/knowledge (2)Perceived competence (3)Quality and utility of training	Follow-up (3 months)	• 97.8% of respondents reported that all domains covered in the training were either helpful or very helpful • 100.0% reported positive changes in perceived competence across 6 of 8 suicide assessment and management skill-based domains • Mean scores for understanding/knowledge domains were all > 90%

The effectiveness of training is outlined below based on the most common domains measured. Thirty studies used questionnaires to explore both the changes as perceived by participants (n = 24) and quantitatively measure change across the different domains (n = 28), while three studies used only interviews to subjectively determine training efficacy with study-specific questions [41–43].

#### Knowledge and attitudes.

Knowledge (n = 23) and attitudes (n = 18) were among the most common domains measured. Statistically significant improvements in total knowledge were reported by 12 studies, of which the training types included three skills-based [39,44,48], three competency-based [53,54,56] and three gatekeeper programs [40,62,63]. The remaining three were non-specific [50], means-restriction [60] and needs- and strengths-based training [36,50,60].

Gryglewicz et al.’s [52] was the only one of seven studies that declared an increase in knowledge of risk factors to produce a significant result (p < 0.001), while Levitt et al. [62] reported a significant increase in knowledge of prevention strategies (p < 0.001). Though Adrian et al. [45] reported increased applied knowledge, and four studies reported improvements in overall suicide prevention knowledge [36,40,44,58], none of these results were statistically significant and, therefore, represent only weak associations. The mixed-method study by Slovak et al. [60] involved a follow-up focus group which outlined that the training increased their knowledge about firearm access, signs and symptoms of suicidality and acquisition of skills and strategies. These findings supported the quantitative findings, where five of the survey items regarding knowledge, strategies and relevance increased significantly (all p < 0.05) from post-test to follow-up.

Three studies declared statistically significant increases in general attitudes towards suicide (all p < 0.001) [36,61,64], and two studies reported a significant reduction in negative attitudes towards suicidal individuals (p = 0.002 and p < 0.001, respectively) [44,49]. Adrian et al. [45] and Hawgood et al. [53] reported significantly increased positive attitudes towards intervention/prevention (p = 0.01 and p = 0.014, respectively) while da Silva Cais [44] reported significant increases for attitudes towards suicide right (p = 0.02). Stuber et al. [48] also reported significant improvement in attitudes to intervention post skills-based training, particularly with regards to conversing about firearms and medication storage (p=<0.01). Cramer et al. [58] declared improvements in 18 of the 21 items on the SBAQ following delivery of a competency-based program; however, significant results were recorded for only 3 subscales: suicide right (p= [0.02); negative attitudes (p = 0.002); and professional capacity (p < 0.001). Additionally, Gryglewicz [49] and colleagues [52], who delivered QPRT gatekeeper training, and Duvivier [47], who delivered a skill-based program and was the only RCT in our sample, did not record significant improvements in attitudes towards suicide prevention.

#### Skills.

Suicide prevention skills were measured by several studies (n = 16), with a number of those reporting overall skill improvement (n = 7); however, only Scott [39], who delivered competency-based training, declared a significant increase (p < 0.000).

Significant improvements were found more specifically in crisis intervention and/or management skills for Gryglewicz et al. [52] and Kawashima et al. [61] (both p < 0.001), and in professional competence (all p < 0.001) for two studies that delivered gatekeeper programs [52,63], three competency-based [54,56,58] and one skills-based training [49]. Risk assessment and/or identification skills were significantly increased post training for Pisani et al. [56], who utilized a documentation exercise to measure change (p < 0.001), and for Jacobson et al. [59], who developed a study-specific questionnaire that included vignette analysis (p = 0.02). Furthermore, two studies declared significant improvements in skills regarding communicating about suicide (both p < 0.001) [36,63], and in two of the qualitative studies [42,43] participants reported improvements to the quality of, and approach towards, discussing suicide with their clients.

In contrast, fifteen studies reported positive results across various domains, including risk assessment (n = 5), crisis management (n = 6) and communication (n = 4). However, improvements were not found to be statistically significant and can thus be considered weak evidence towards skill development in those areas.

#### Confidence and self-efficacy.

Confidence (n = 13) and self-efficacy (n = 11) were measured by several studies, typically exploring participants’ level of comfort with, and understanding of, approaching suicide with their clients and their responses to suicidal behaviors. The greatest increases were found in perceived capacity and/or skill in managing suicidal individuals, including communication and de-escalation of crises, with five studies declaring significant results (all p < 0.001) [35,40,48,54,61]. Only one study reported significant results for increased confidence in identifying suicide warning signs and safety planning (p=<0.01) [48]. In addition, only one study identified increased confidence in responding to suicidal behavior (p = 0.008) [54]. Two studies [52,53] found improvements in participants’ perceived capability post training, though results were significant in only one of the two studies [53]. It is also worth noting that whilst Hawgood et al. detected a significant change in perceived capability, participants’ reluctance to intervene did not significantly improve, which indicates mixed evidence regarding changes in confidence and self-efficacy.

All three qualitative studies discussed improvements in confidence and self-efficacy. Gask et al. [41] reported that the skills-based training improved participants’ confidence in identifying risks and asking patients about suicidal behaviors, as well as discussing protective factors and problem solving. Jones et al. [42] reported their participants felt increased confidence working with those at risk and were better equipped to manage suicidality, particularly their responses to disclosure of self-harm and suicidal ideation. Furthermore, the participants from Mirick et al. [43], who delivered competency-based training, described feeling more comfortable in communicating about suicide and, specifically, in using more direct language and/or adjusting their questions to elicit more truthful responses.

#### Practice changes.

Fourteen studies measured the degree of change or intent of participants to implement learning in their clinical practice. This was typically done in the form of “yes/no” questions or using a Likert scale. A range of 43–97% of participants from 9 of the 13 studies that conducted follow-up reported implementation of training knowledge or skills [39,41–43,46,49,50,57,59], including changes to client management and communication. Notably, 97% of participants from Wharff et al.’s [57] study reported a substantial increase in the level of inquiry when conducting risk assessments, stating that they no longer took their client’s negative responses to questions about suicidality at “face value”.

Improved information exchange was also declared by Adrian et al. [45], Mirick et al. [43,55] and Slovak et al. [60], and increased referral to services by Condron et al. [37] and Huh et al. [46]. Participants from the qualitative study by Mirick et al. [43] described how they increased their focus on protective factors, conducted more in-depth risk assessments, and increased the rate of safety planning with their clients post-training.

#### Other.

Overall, there were no significant differences in results between the training delivery modes or duration, with many of the brief trainings producing similar results to the longer trainings. However, the two studies that compared brief and enhanced training did report large differences between the two groups, with greater gains achieved by the longer workshops. Condron et al. [37] reported significant differences in risk identification (p < 0.001) and referral to services (p = 0.009) for the enhanced training group, while Donald et al. [50] outlined significant increases in knowledge, information exchange and support (all p ≤ 0.001).

Seven studies did not obtain a baseline measurement [37,41–43,57,59,60]. Six studies reported maintenance of results at follow-up (T3), with only small decreases in outcomes compared to post-test [45,46,49,50,59,62]. Furthermore, prior exposure to suicide prevention training did not seem to moderate learning effects, with only two studies noting an impact [55,61].

## Discussion

The objective of this scoping review was to provide a synthesis of the literature surrounding suicide prevention training delivered to AHPs in health care environments. To our knowledge, no other review has specifically addressed this topic. Since the commencement of this research, at least two systematic reviews have been published addressing suicide prevention training [65,66]; however, both examined gatekeeper programs that were delivered exclusively to family and friends of at-risk individuals or to general community members. The 33 studies reviewed in this study reported on an array of training types, methodological approaches, and outcomes; however, no clear patterns of best practice were discernible, and the evidence of training effectiveness was generally weak.

Despite the absence of limiters applied during the search and selection processes, all but one of the included studies were conducted in high-income countries, and two-thirds of those were from the United States. While this may be due to a lack of research production in low- and middle-income countries, it has been argued that a substantial part of the world is overlooked for scientific contributions in what is known as geographic bias [67]. Though it is beyond the scope of this review to comprehensively analyze the biases surrounding peer-review publication, it is important to note the lack of population diversity, both in terms of culture and socio-economic status, that questions the transferability of findings and impacts generalizability of the results internationally. Furthermore, when exploring capacity building options for practitioners in the support of suicide prevention, health research needs to be translational to inform and generate international best practice for the global suicide epidemic. The narrow scope of training found in this review represents additional barriers to informing international practice.

Additionally, the majority of included papers had target populations either exclusive to, or largely inclusive of, health professionals more generally, with only a limited number of studies on suicide prevention training specifically targeting AHPs. This indicates the training content is not purposely designed for the allied health professions and may potentially be less effective when delivered to allied health disciplines. Given the diversity and nuances between the allied health professions, a deeper understanding of how each discipline can contribute to the efforts of suicide prevention and how the training programs should be tailored to best support AHPs is warranted.

A personalized approach to suicide prevention training is essential to ensure that AHPs receive the knowledge and skills most relevant to their practice and the nature of engagement with their patients. For example, physiotherapists may need training that incorporates strategies for recognizing suicidal behavior in patients with chronic pain, while speech pathologists may benefit from learning how to identify communication barriers that could signal distress. Tailoring training to address the specific ways each profession interacts with at-risk individuals could enhance both the effectiveness of the interventions and the confidence of AHPs in their suicide prevention efforts.

To implement such personalization, training programs could:

1) Conduct Needs Assessments – Begin with a thorough assessment of the specific needs and challenges of each allied health profession in relation to the nature of engagement with their specific patients/clients/consumers. Surveys, focus groups, or interviews with practitioners can help identify the unique situations and stressors they encounter.
2) Customize Content by Discipline – Develop training modules that reflect the distinct roles and responsibilities of different AHPs. For instance, modules could be designed to address the physical, psychological, and social factors that AHPs in different fields regularly manage, ensuring the content is relevant to their practice. Matching training to identified, discipline-specific scope of practice.
3) Use Case-Based Learning – Incorporate case studies and role-playing scenarios that are specific to each discipline. These real-world examples can help AHPs better understand when and how to apply suicide prevention strategies, within their practical care pathway as part of their day-to-day practice, making the training more practical, engaging and translatable.
4) Offer Flexible Delivery Modes – Provide options for how the training is delivered, such as in-person workshops, online modules, or hybrid formats, to accommodate different learning preferences and workplace demands. Flexibility in program delivery allows for training that fits within the schedules and constraints of various professions and contexts, and reduces barriers to engaging in important capacity building opportunities.

By personalizing suicide prevention training to meet the specific needs of different AHPs, we can enhance the overall effectiveness of the programs, ensuring they equip professionals with the right tools to support suicide prevention efforts in their respective fields.

The participants of the included training programs were predominantly female, which was notable but unsurprising given that females form approximately 70% of the global health care workforce [3], and account for almost twice the number of male allied health professionals in both Australia and the United States [68,69]. Interestingly, most participants were over 35 years of age, which is curious as a considerable number of health practitioners are aged 20–34, and annual CPD requirements are largely mandated for those in allied health upon professional registration, which usually takes place following completion of tertiary studies [70]. This raises questions as to the targeting and promotion of suicide prevention training for younger clinicians, and further investigation is needed to understand the reasons for this disparity as such training is important for practitioners of all levels.

The duration of the training programs varied widely and ranged from 1–20 hours; however, the mode of delivery was reasonably consistent. Although there is strong evidence that online training is as effective and at times superior to instructor-led, face-to-face training [71,72], almost all of the included studies delivered face-to-face workshops and only one adopted a blended learning approach [49]. It is known that web-based instruction is more accessible, less expensive and requires fewer overall resources than face-to-face learning [71]. Increasing the availability of online programs could considerably improve accessibility of suicide prevention training to AHPs in lower income countries or when resources and time constraints are factors in large health services worldwide.

Of the studies that specifically targeted AHPs, participants were more likely to work in the field of mental health and therefore undertook the training with greater baseline knowledge, potentially influencing study results. In addition, the studies targeting AHPs largely consisted of social workers as the only allied health discipline, though one study was designed for pharmacists. As discussed earlier, exposure to suicidality is not limited to specific health disciplines and it is imperative that all AHPs maintain and update their skills and knowledge throughout their careers [12,73]. Thus, the lack of diversity in allied health professions being targeted for suicide prevention training reveals a gap in the delivery of such programs.

The types of training also varied greatly; however, skills-based, gatekeeper and competency-based programs were the most common. Skills-based programs tend to focus on the development of risk assessment and/or crisis management skills, as well as effective communication [4]. Three of the nine studies that delivered skills-based programs showed statistically significant results across the various domains measured, with significant improvements found for risk identification and assessment, eliciting protective factors and safety planning, as well as attitudes towards intervention, reduced negativity towards suicidality and professional competencies [44,48,49]. However, eight studies that delivered other training types also declared statistically significant results within the same domains, and therefore this cannot be considered strong evidence towards skill-based programs specifically at this stage.

Gatekeeper training is widely used and reportedly effective across a range of populations and settings including schools, communities, and businesses [74,75], though there is less evidence to support meaningful behavior change within healthcare [20,76]. The studies that delivered gatekeeper training reported statistically significant results in the categories of knowledge, attitudes, and skills, though these were across limited domains and lacked a clear pattern to determine a causal relationship. Additionally, only three of the eight gatekeeper studies were targeted specifically at AHPs, further weakening the generalizability of the results for this population.

Professional competencies consist of the knowledge, skills and attributes that are required to work in a particular field. For health professionals, it is common for the governing board of each discipline to have individualized competencies outlined that guide professional conduct [77].The competency-based programs included in this review were designed to address the core competencies of suicide risk assessment and management, including knowledge of risk/protective factors, managing personal attitudes and reactions to suicidal behaviors, providing empathetic care, formulating safety plans and maintaining appropriate documentation. Of the eight studies delivering competency-based training, four reported statistically significant improvements [39,53,54,56], though these results were mostly for subjective domains such as perceived professional competence and perceived ability, and thus may not truly reflect meaningful change in suicide prevention practice.

The diversity of the frameworks, models and approaches used in the included studies was even greater than the types of training. Within the different training types there were multiple programs delivered, with the greatest heterogeneity amongst the skills-based frameworks. However, approximately 40% of the studies designed the training content specifically for the study and did not adopt a previously developed and/or validated training program. Several key constructs were consistent amongst the diverse approaches; for example, content surrounding suicide prevention knowledge was found in every study. Conversely, three studies had specific content regarding counselling on lethal means and one involving CBT [39,59,60], which further increased the variety of what was delivered. There were no particular frameworks that stood out from the others regarding outcomes, with many of the different programs achieving some statistically significant results. While this indicates that a variety of training models may be useful and/or effective across a number of constructs, clear evidence of best practice remains inconclusive.

Similarly, the types of measures and specific measurement tools used were largely heterogenous, making generalizability of results difficult. As with the training content, many used instruments designed for the particular study and/or adapted existing tools to study needs. While this is acceptable practice in research, it potentially compromises the reliability and/or validity of a measurement tool and the subsequent results [78]. In addition, approximately 80% of the studies conducted their post-test immediately upon completion of the training, increasing the possibility of a positive outcome and/or biased representation of learning transfer [79]. Less than half of the studies conducted follow-up measures and only half of those reported maintenance of learning. Often this was determined by subjective questions rather than a quantifiable measure and may therefore be considered less reliable as an indication of change [80].

### Limitations

As it is not typically performed in scoping reviews, a quality appraisal of the evidence was not conducted. Similarly, a risk of bias assessment was not conducted as it is not applicable to the JBI framework utilized in this study. Thus, this review was also limited by publication bias, whereby published research most often declares positive outcomes. Despite the ever-growing issue of publication bias [81], this review presented an array of noteworthy results and outlined some important limitations of the included research.

The heterogeneity of the training types and underpinning frameworks, in addition to the diverse measures used in the included studies, made it challenging to compare the outcomes. The analysis was further limited by the lack of diversity in country of origin. However, these are gaps in current knowledge that this review has identified, facilitating the development and focus of future research in this area.

## Conclusions

It is well established that global efforts toward reduction of deaths by suicide are supported by a well-trained workforce. Therefore, investing in efficacious training and professional development opportunities is key to building the capacity to reduce suicide mortality in our community. However, it is equally important to ensure the training approach best supports the scope of practice of the health professional and research is conducted with a lens of diversity and inclusion to ensure generalizability. Synthesis of the current evidence was challenged in this review by the heterogeneity of the results, and though overall positive outcomes from suicide prevention training programs were broadly reported, strong evidence of meaningful change in most circumstances was weak. Moreover, international application of the current evidence is difficult due to the lack of diversity in study origin, and additional investigation into suicide prevention training particularly outside of the United States is required to provide better understanding of current training practices internationally.

### Implications for research

This review has identified significant gaps in knowledge of best practice within the field of allied health and outlined areas of enquiry that can improve the future delivery of suicide prevention training. Moreover, the merit to doing this with consideration of nuances within the disciplines making up the allied health professions was also identified. It is recommended that further research is undertaken to better determine the efficacy of the current programs available, suitably informing and supporting future suicide prevention practice by AHPs in health care environments.

## Supporting information

S1 Appendix Search Strategy. (DOCX)

S2 Appendix Studies Ineligible Following Full-Text Review. (DOCX)

S3 Appendix Complete Summary Table. (DOCX)
