# Supplementary material for: Suicide prevention training for allied health professionals within healthcare environments: A scoping review
Source: PLoS One. 2025 Aug 8;20(8):e0326738. doi: 10.1371/journal.pone.0326738 (PMC12334045; doi:10.1371/journal.pone.0326738)
Supplement: S1 Appendix — (DOCX) [file pone.0326738.s001.docx]

**Complete Search Strategy for CINAHL (EBSCOhost) – Conducted 07/04/2021**

| Search # | Search terms | Search Options | Results |
| --- | --- | --- | --- |
| S1 | (MH "Occupational Therapists") OR (MH "Physical Therapists") OR (MH "Social Workers") OR (MH "Mental Health Personnel") OR (MH "Psychologists") OR (MH "Psychotherapists") OR (MH "Nurses") OR (MH "Psychiatrists") OR (MH "Physicians, Emergency") OR (MH "Physicians") OR (MH "Health Personnel") OR (MH "Physicians, Family") | **Expanders –** Apply equivalent subjects  **Search modes –** Boolean/Phrase | 236,600 |
| S2 | TI (health professional* or physician* or general practitioner* or nurs* or social work* or occupational therapist* or physiotherapist* or psychologist* or psychiatrist*) OR AB (health professional* or physician* or general practitioner* or nurs* or social work* or occupational therapist* or physiotherapist* or psychologist* or psychiatrist*) | **Expanders –** Apply equivalent subjects  **Search modes –** Boolean/Phrase | 828,450 |
| S3 | (MH "Suicide") OR (MH "Suicide, Attempted") OR (MH "Suicidal Ideation") | **Expanders –** Apply equivalent subjects  **Search modes –** Boolean/Phrase | 29,833 |
| S4 | TI (suicid* N3 (prevent* or risk assess* or control or attempted or completed or ideation or behavio#r)) OR AB (suicid* N3 (prevent* or risk assess* or control or attempted or completed or ideation or behavio#r)) | **Expanders –** Apply equivalent subjects  **Search modes –** Boolean/Phrase | 14,770 |
| S5 | (MH "Refresher Courses") OR (MH "Education, Continuing") | **Expanders –** Apply equivalent subjects  **Search modes –** Boolean/Phrase | 13,718 |
| S6 | TI ((continuing or professional) N3 educat*) or train* or inservice training) OR AB ((continuing or professional) N3 educat*) or train* or inservice training) | **Expanders –** Apply equivalent subjects  **Search modes –** Boolean/Phrase | 269,477 |
| S7 | S1 OR S2 | **Expanders –** Apply equivalent subjects  **Search modes –** Boolean/Phrase | 958,438 |
| S8 | S3 OR S4 | **Expanders –** Apply equivalent subjects  **Search modes –** Boolean/Phrase | 33,186 |
| S9 | S5 OR S6 | **Expanders –** Apply equivalent subjects  **Search modes –** Boolean/Phrase | 279,060 |
| S10 | S7 AND S8 AND S9 | **Limiters –** Published Date: 20100101-20210407  **Expanders –** Apply equivalent subjects  **Search modes –** Boolean/Phrase | 483 |

**Complete Search Strategy for CINAHL (EBSCOhost) – Conducted 10/07/2023**

| **Search #** | **Search terms** | **Search Options** | **Results** |
| --- | --- | --- | --- |
| **S1** | TI (“health professional*” OR “allied health” OR “social work*” OR “occupational therap*” OR physiotherap* OR “physical therap*” OR pharmac* OR nutrition* OR “speech patholog*” OR diet* OR paramedic* OR podiatr* OR osteopath* OR (professional* N3 "mental health")) OR AB (“health professional*” OR “allied health” OR “social work*” OR “occupational therap*” OR physiotherap* OR “physical therap*” OR pharmac* OR nutrition* OR “speech patholog*” OR diet* OR paramedic* OR podiatr* OR osteopath* OR (professional* N3 "mental health")) | **Expanders –** Apply equivalent subjects  **Search modes –** Boolean/Phrase | 567, 351 |
| **S2** | TI ((suicid* prevent* OR suicid* risk assess* OR suicid* control) N6 (training OR education)) OR AB ((suicid* prevent* OR suicid* risk assess* OR suicid* control) N15 (training OR education)) | **Expanders –** Apply equivalent subjects  **Search modes –** Boolean/Phrase | 589 |
| **S3** | (MH "Health Personnel") OR (MH “Health Personnel, Education”) OR (MH “Allied Health Personnel”) OR (MH “Alternative Health Personnel”) OR (MH "Mental Health Personnel") OR (MH “Multidisciplinary Care Team”) OR (MH "Social Workers") OR (MH "Occupational Therapists") OR (MH "Physical Therapists") OR (MH Pharmacists) OR (MH “SpeechLanguage Pathologists”) OR (MH Dietitians) OR (MH “Emergency Medical Technicians”) OR (MH Podiatrists) OR (MH Osteopaths) | **Expanders –** Apply equivalent subjects  **Search modes –** Boolean/Phrase | 184, 883 |
| **S4** | MH "Suicide") OR (MH "Suicide, Attempted") OR (MH "Suicidal Ideation") OR (MH "Refresher Courses") OR (MH "Education, Continuing") OR (MH “Education, Emergency Medical Services”) | **Expanders –** Apply equivalent subjects  **Search modes –** Boolean/Phrase | 46, 032 |
| **S5** | S1 OR S3 | **Expanders –** Apply equivalent subjects  **Search modes –** Boolean/Phrase | 696, 742 |
| **S6** | S2 OR S4 | **Expanders –** Apply equivalent subjects  **Search modes –** Boolean/Phrase | 46, 305 |
| **S7** | S5 AND S6 | **Limiters -** Published Date: 20220601- 20230731 **Expanders -** Apply equivalent subjects  **Search modes -** Boolean/Phrase | 272 |
