# Supplementary material for: Suicide prevention training for allied health professionals within healthcare environments: A scoping review
Source: PLoS One. 2025 Aug 8;20(8):e0326738. doi: 10.1371/journal.pone.0326738 (PMC12334045; doi:10.1371/journal.pone.0326738)
Supplement: S2 Appendix — (DOCX) [file pone.0326738.s002.docx]

**S2: Studies ineligible following full-text review**

1. Allman F, Lee-Savage H. Development of a suicide intervention training programme for secondary mental health services. Mental Health Practice. 2020;23(2):22-8.

Reason for exclusion: non-empirical study.

1. Almeida J, O'Brien KHM, Gironda CM, Gross EB. Development, implementation, and evaluation of a comprehensive course on suicide in a master’s of social work program. Journal of Social Work Education. 2017;53(4):727-36.

Reason for exclusion: training delivered to non-allied health professionals.

1. Alonzo D, Popescu M, Zubaroglu-Ioannides P. Effects of brief training on mental health provider's knowledge of working with youth at risk of suicide in Guatemala. International Journal of Social Psychiatry. 2022;68(2):281-287.

Reason for exclusion: Community or school-based setting.

1. Atuel HR, Kintzle S. Comparing the training effectiveness of virtual reality and role play among future mental health providers. *Psychological Trauma: Theory, Research, Practice, and Policy*. 2021,13(6):657-664.

Reason for exclusion: training delivered to non-allied health professionals.

1. Awyie, CA. Staff education to improve knowledge about suicide screening. ProQuest Information & Learning, 2023.

Reason for exclusion: Training delivered to non-allied health professionals.

1. Boukouvalas EA, El-Den S, Chen TF, Moles R, Saini B, Bell A, et al. Confidence and attitudes of pharmacy students towards suicidal crises: patient simulation using people with a lived experience. *Social Psychiatry & Psychiatric Epidemiology*. 2018;53(11):1185-95.

Reason for exclusion: training delivered to non-allied health professionals.

1. Breux P, Boccio DE. Improving Schools' Readiness for Involvement in Suicide Prevention: An Evaluation of the Creating Suicide Safety in Schools (CSSS) Workshop. *International Journal of Environmental Research & Public Health*. 2019;16(12):19.

Reason for exclusion: community or school-based setting.

1. Brown JA, Goforth AN, Machek G. School psychologists’ experiences with and training in suicide assessment: Challenges in a rural state. *Contemporary School Psychology*. 2018;22(2):195-206.

Reason for exclusion: training delivered to psychologists or counsellors only.

1. Brown RC, Straub J, Bohnacker I, Plener PL. Increasing knowledge, skills, and confidence concerning students’ suicidality through a gatekeeper workshop for school staff. *Frontiers in Psychology*. 2018;9.

Reason for exclusion: training delivered to psychologists or counsellors only.

1. Caine ED, Cross WF. What would it take to prepare health care professionals to work with suicidal persons? *American Journal of Public Health*. 2018;108(6):717-8.

Reason for exclusion: non-empirical study.

1. Carpini, J A, Sharma, A, Kubicki Evans M, et al. Pharmacists and Mental Health First Aid training: A comparative analysis of confidence, mental health assistance behaviours and perceived barriers. *Early Intervention in Psychiatry*. 2022;17(7):270-280.

Reason for exclusion: Non-empirical study.

1. Chauliac N, Brochard N, Payet C, Margue Y, Bordin P, Depraz P, et al. How does gatekeeper training improve suicide prevention for elderly people in nursing homes? A controlled study in 24 centres. *European Psychiatry*. 2016;37:56-62.

Reason for exclusion: community or school-based setting.

1. Cochran CB. An evidence-based approach to suicide risk assessment after sexual assault. *Journal of Forensic Nursing*. 2019;15(2):84-92.

Reason for exclusion: training delivered to non-allied health professionals.

1. Colucci E, Jaroudy S, Rossmann M. Piloting of a suicide first aid gatekeeper training (online) for children and young people in conflict affected areas in Syria. *International Review of Psychiatry*. 2022;34(6):640-648.

Reason for exclusion: Training delivered to non-allied health professionals.

1. Conner KR, Wood J, Pisani AR, Kemp J. Evaluation of a suicide prevention training curriculum for substance abuse treatment providers based on Treatment Improvement Protocol Number 50. *Journal of Substance Abuse Treatment*. 2013;44(1):13-6.

Reason for exclusion: training delivered to psychologists or counsellors only.

1. Coppens E, Van Audenhove C, Gusmão R, Purebl G, Székely A, Maxwell M, et al. Effectiveness of General Practitioner training to improve suicide awareness and knowledge and skills towards depression. *Journal of Affective Disorders*. 2018;227:17-23.

Reason for exclusion: community or school-based setting.

1. Cramer RJ, La Guardia AC, Wright-Berryman J, Long MM, Adams Tufts K. Integrating Interprofessional Education into Suicide Prevention Training: Results from A Pilot Evaluation. *Social Work in Public Health*. 2019;34(7):628-36.

Reason for exclusion: training delivered to non-allied health professionals.

1. De Beurs DP, Bosmans JE, De Groot MH, De Keijser J, Van Duijn E, De Winter RFP, et al. Training mental health professionals in suicide practice guideline adherence: Cost-effectiveness analysis alongside a randomized controlled trial. *Journal of Affective Disorders*. 2015;186:203-10.

Reason for exclusion: non-empirical study.

1. De Beurs DP, De Groot MH, De Keijser J, Van Duijn E, De Winter RFP, Kerkhof AJFM. Evaluation of benefit to patients of training mental health professionals in suicide guidelines: Cluster randomised trial. *British Journal of Psychiatry*. 2016;208(5):477-83.

Reason for exclusion: non-empirical study.

1. De Silva E, Bowerman L, Zimitat C. A suicide awareness and intervention program for health professional students. *Education for Health: Change in Learning & Practice*. 2015;28(3):201-4.

Reason for exclusion: training delivered to non-allied health professionals.

1. Donovan S, Maggiulli L, Aiello J, Centeno P, John S, Pisani A. Evaluation of sustainable, blended learning workforce education for suicide prevention in youth services. Children and Youth Services Review. 2023;148:1-13.

Reason for exclusion: Community or school-based setting.

1. Elzinga E, de Kruif A, de Beurs DP, Beekman ATF, Franx G, Gilissen R. Engaging primary care professionals in suicide prevention: A qualitative study. *PLoS ONE*. 2020;15(11):e0242540.

Reason for exclusion: training delivered to non-allied health professionals.

1. Faria JS, Marcon SR, Nespollo, AM, et al. Attitudes of health professionals towards suicidal behavior: An intervention study. *Revista de Saude Publica*. 2022;56(54).

Reason for exclusion: Training delivered to non-allied health professionals.

1. Ferguson M, Dollman J, Jones M, Cronin K, James L, Martinez L, et al. Suicide prevention training—Improving the attitudes and confidence of rural Australian health and human service professionals. *Crisis: The Journal of Crisis Intervention and Suicide Prevention*. 2019;40(1):15-26.

Reason for exclusion: community or school-based setting.

1. Flynn D, Joyce M, Weihrauch M, O'Malley C. DBT STEPS-A: Inter-agency collaboration to promote positive mental health in adolescents. *International Journal of Integrated Care (IJIC*). 2017;17:1-2.

Reason for exclusion: community or school-based setting.

1. Gask L, Coupe N, Green G. An evaluation of the implementation of cascade training for suicide prevention during the 'Choose Life' initiative in Scotland - utilizing Normalization Process Theory. *BMC Health Services Research*. 2019;19(1):N.PAG-N.PAG.

Reason for exclusion: training delivered to non-allied health professionals.

1. Higgins J. Integrated Services and Suicide Prevention Training. *Professional Case Management.* 2014;19(3):137-42.

Reason for exclusion: non-empirical study.

1. Jones R. The development of nurse-led suicide prevention training for multidisciplinary staff in a North Wales NHS Trust. *Journal of Psychiatric and Mental Health Nursing*. 2010;17(2):178-83.

Reason for exclusion: non-empirical study.

1. Kaniwa I, Kawanishi C, Suda A, Hirayasu Y. Effects of educating local government officers and healthcare and welfare professionals in suicide prevention. *International Journal of Environmental Research & Public Health*. 2012;9(3):712-21.

Reason for exclusion: training delivered to non-allied health professionals.

1. Kresin T, & Forster EM. Suicide awareness training: A literature review with application to Queensland nurses. *Issues in Mental Health Nursing*. 2022;43(1);13-21.

Reason for exclusion: Training delivered to non-allied health professionals.

1. Kullberg MLJ, Mouthaan J, Schoorl M, De Beurs D, Kenter RMF, Kerkhof AJFM. E-learning to improve suicide prevention practice skills among undergraduate psychology students: Randomized controlled trial. *JMIR Mental Health*. 2020;7(1).

Reason for exclusion: training delivered to non-allied health professionals.

1. Lavigne J, Cross W., Shook A, Carpenter D. O.28.2 - Co-developing a suicide prevention training module with community pharmacy staff: Presenter(s): Amanda Stover, University of North Carolina, Eshelman School of Pharmacy, United States. *Patient Education & Counseling.* 2023;109:115.

Reason for exclusion: Non-empirical study.

1. Little V, James MC. Suicide Safer Care: A Public Health Approach to Training Primary Care Providers in Addressing Suicide. *Journal of Health Care for the Poor & Underserved*. 2020;31(3):1050-3.

Reason for exclusion: full-text unavailable.

1. Lofving Gupta S, Wijk K, Warner G, Sarkadi A. Readiness of Allied Professionals to Join the Mental Health Workforce: A Qualitative Evaluation of Trained Lay Trauma Counsellors' Experiences When Refugee Youth Disclose Suicidal Ideation. *International Journal of Environmental Research & Public Health*. 2021;18(4):04.

Reason for exclusion: non-empirical study.

1. LoParo D, Florez IA, Valentine N, Lamis DA. Associations of Suicide Prevention Trainings with Practices and Confidence among Clinicians at Community Mental Health Centers. *Suicide & Life-Threatening Behavior*. 2019;49(4):1148-56.

Reason for exclusion: training delivered to non-allied health professionals.

1. Maciag R, Travers‐Hill E, Morrison, NR, & Kim Y. Paraprofessionals delivering brief psychological interventions: Qualitative accounts of training and supervision. *Counselling & Psychotherapy Research*. 2023;23(1):64-73.

Reason for exclusion: Training delivered to non-allied health professionals.

1. Magaletta PR, McLearen AM. Clinical supervision in prison settings: Three strategies for approaching suicide risk. *Journal of Aggression, Conflict and Peace Research*. 2015;7(3):149-57.

Reason for exclusion: non-empirical study.

1. Marshall E, York J, Magruder K, Yeager D, Knapp R, Santis ML, et al. Implementation of online suicide-specific training for VA providers. *Academic Psychiatry*. 2014;38(5):566-74.

Reason for exclusion: training delivered to non-allied health professionals.

1. Matthieu MM, Hensley MA. Gatekeeper training outcomes: Enhancing the capacity of staff in substance abuse treatment programs to prevent suicide in a high risk population. *Mental Health and Substance Use*. 2013;6(4):274-86.

Reason for exclusion: full-text unavailable.

1. Matthieu MM, Swensen AB. Suicide prevention training program for gatekeepers working in community hospice settings. *Journal of Social Work in End-of-Life & Palliative Care*. 2014;10(1):95-105.

Reason for exclusion: community or school-based setting.

1. McLaughlin S, Bonner G, Canning C. Improving confidence in suicide risk assessment. *Nursing Times*. 2014;110(27):16-8.

Reason for exclusion: non-empirical study.

1. Mitchell SM, Taylor NJ, Jahn DR, Roush JF, Brown SL, Ries R, et al. Suicide-Related Training, Self-Efficacy, and Mental Health Care Providers' Reactions Toward Suicidal Individuals. *Crisis: Journal of Crisis Intervention & Suicide*. 2020;41(5):359-66.

Reason for exclusion: training delivered to psychologists or counsellors only.

1. Mueller KL, Chirumbole D, Naganathan S. Counseling on Access to Lethal Means in the Emergency Department: A Script for Improved Comfort. *Community Mental Health Journal.* 2020;56(7):1366-71.

Reason for exclusion: non-empirical study.

1. Ng R, O'Reilly CL, Collins JC, et al. Mental Health First Aid crisis role-plays between pharmacists and simulated patients with lived experience: A thematic analysis of debrief. *Social Psychiatry and Psychiatric Epidemiology*. 2023;16.

Reason for exclusion: Community or school-based setting.

1. O'Driscoll M, Carpenter, DM, Foley A, Moloney E, Reddin K, Sahm LJ. A needs assessment for suicide prevention training within community pharmacies. *Exploratory Research in Clinical and Social Pharmacy*. 2023;10:e100285.

Reason for exclusion: Community or school-based setting.

1. Osteen P, Frey JM, Woods MN, Ko J, Shipe S. Modeling the longitudinal direct and indirect effects of attitudes, self-efficacy, and behavioral intentions on practice behavior outcomes of suicide intervention training. *Suicide & Life-Threatening Behavior.* 2017;47(4):410-20.

Reason for exclusion: non-empirical study.

1. Osteen PJ. Suicide intervention gatekeeper training: Modeling mediated effects on development and use of gatekeeper behaviors. *Research on Social Work Practice*. 2018;28(7):848-56.

Reason for exclusion: training delivered to non-allied health professionals.

1. Pierce D, Little F, Bennett-Levy J, Isaacs AN, Bridgman H, Lutkin SJ, et al. Mental health academics in rural and remote Australia. *Rural & Remote Health*. 2016;13(3):1-11.

Reason for exclusion: non-empirical study.

1. Robles R, Lopez-Garcia P, Miret M, Cabello M, Cisneros E, Rizo A, et al. WHO-mhGAP training in Mexico: Increasing knowledge and readiness for the identification and management of depression and suicide risk in primary care. *Archives of Medical Research*. 2019;50(8):558-66.

Reason for exclusion: training delivered to non-allied health professionals.

1. Roslan A F, Pheh KS, Mahadevan R, et al. Effectiveness of online advanced C.A.R.E suicide prevention gatekeeper training program among healthcare lecturers and workers in national university of Malaysia: A pilot study. *Frontiers in Psychiatry*. 2023;14.

Reason for exclusion: Training delivered to non-allied health professionals.

1. Ryan K, Tindall C, Strudwick G. Enhancing key competencies of health professionals in the assessment and care of adults at risk of suicide through education and technology. *Clinical Nurse Specialist: The Journal for Advanced Nursing Practice*. 2017;31(5):268-75.

Reason for exclusion: non-empirical study.

1. Santos JC, Simões RMP, De Azevedo Erse MPQ, Façanha JDN, Marques LAFA. Impact of “+contigo” training on the knowledge and attitudes of health care professionals about suicide. *Revista Latino-Americana de Enfermagem*. 2014;22(4):679-84.

Reason for exclusion: community or school-based setting.

1. Sharpe TL, Jacobson Frey J, Osteen PJ, Bernes S. Perspectives and appropriateness of suicide prevention gatekeeper training for MSW students. *Social Work in Mental Health.* 2014;12(2):117-31.

Reason for exclusion: training delivered to non-allied health professionals.

1. Siau CS, Wee LH, Ibrahim N, Visvalingam U, Yeap LLL, Wahab S. Gatekeeper suicide training's effectiveness among Malaysian hospital health professionals: A control group study with a three-month follow-up. *Journal of Continuing Education in the Health Professions*. 2018;38(4):227-34.

Reason for exclusion: training delivered to non-allied health professionals.

1. Silva C, Smith AR, Dodd DR, Covington DW, Joiner TE. Suicide-related knowledge and confidence among behavioral health care staff in seven states. *Psychiatric Services*. 2016;67(11):1240-5.

Reason for exclusion: full-text unavailable.

1. Smith AR, Silva C, Covington DW, Joiner JTE. An assessment of suicide-related knowledge and skills among health professionals. *Health Psychology.* 2014;33(2):110-9.

Reason for exclusion: training delivered to non-allied health professionals.

1. Smith LH. Suicide and depression education provided in doctorate of physical therapy programs: ProQuest Information & Learning; 2011.

Reason for exclusion: training delivered to non-allied health professionals.

1. Stover AN, Lavigne JE, Carpenter, DM. A scoping review of suicide prevention training programs for pharmacists and student pharmacists. *American Journal of Pharmaceutical Education*. 2023;87(3):ajpe8917.

Reason for exclusion: Non-empirical study.

1. Stover, AN, Lavigne JE, Shook A, MacAllister C, Cross WF, Carpenter, DM. Development of the Pharm‐SAVES educational module for gatekeeper suicide prevention training for community pharmacy staff. *Health Expectations*. 2023;26(3):1246-1254.

Reason for exclusion: Non-empirical study.

1. Suldo S, Loker T, Friedrich A, Sundman A, Cunningham J, Saari B, et al. Improving school psychologists' knowledge and confidence pertinent to suicide prevention through professional development. *Journal of Applied School Psychology*. 2010;26(3):177-97.

Reason for exclusion: training delivered to psychologists or counsellors only.

1. Viard, MC, Grandgenevre P, Bubrovszky, M, et al. Impact of the suicidal crisis intervention training program on the confidence and skills of hospital professionals in the Hauts-de-France region. *Encephale*. 2022;49(5):504-509.

Reason for exclusion: Training delivered to non-allied health professionals.

1. Wand APF, Jessop T, Peisah C. Educating crisis supporters about self-harm and suicide in older adults. *The American Journal of Geriatric Psychiatry*. 2022;30(11):1212-1220.

Reason for exclusion: Training delivered to non-allied health professionals.

1. Weatherbee MJ. The impact of a brief training on suicide for graduate students in psychology, incorporating Thomas Joiner's theory of why people die by suicide: ProQuest Information & Learning; 2011.

Reason for exclusion: training delivered to psychologists or counsellors only.

1. Witry MJ, Neblett K, Hutchens S, Catney C. When a patient talks about suicide: Adding a social worker led session on the pharmacist's role in suicide prevention to the PharmD curriculum. *Currents in Pharmacy Teaching & Learning*. 2019;11(6):585-91.

Reason for exclusion: training delivered to non-allied health professionals.

1. Wong KTN. Perceived competence in suicide intervention and attitudes towards suicide among mental health professionals and trainees in Hong Kong: ProQuest Information & Learning; 2018.

Reason for exclusion: non-empirical study
