# Supplementary material for: Suicide prevention training for allied health professionals within healthcare environments: A scoping review
Source: PLoS One. 2025 Aug 8;20(8):e0326738. doi: 10.1371/journal.pone.0326738 (PMC12334045; doi:10.1371/journal.pone.0326738)
Supplement: S3 Appendix — (DOCX) [file pone.0326738.s003.docx]

| **Author(s)**  **Year & Country** | **Study design and Aim** | **Study population and sample size** | **Type of training** | **Delivery mode and duration** | **Theoretical framework** | **Measures** | **Outcomes** |
| --- | --- | --- | --- | --- | --- | --- | --- |
| Adrian et al. (2018)  USA | Case Series  This study examined the impact of a 6-hr continuing education training, and the effect of a post training reminder system, on mental health practitioners’ knowledge, attitudes, and behavior surrounding suicide assessment and intervention. | 83 health practitioners working at Seattle Children’s Hospital (SCH) - 75 completed evaluations (~10% drop-out rate) 29 psychologists (34.9%); 25 social workers (30.1%); 14 mental health therapists (16.9%); 4 paediatric mental health specialists (4.8%); 11 other (13.2%).   82.9% were Female  *ages not provided | Skill-based:  Suicide risk assessment and management training, designed by a team of psychologists/psychiatrists tailored specifically for SCH | 6-hour in person training workshop at SCH | Non-specific:  Training comprised four content areas:  (1) descriptive epidemiology,  (2) assessment strategies, (3) management of suicide risk  (4) special populations and cultural considerations. | Pre; Post (immediate); Follow-up (3 months)  *-Suicide Interventionist Response Inventory (SIRI-2)*  -*Attitudes Toward Standard Assessment*  *Scales-Adapted (ASA)*  *-Suicide Intervention Questionnaire (SIQ)* | -Significant increases were found in suicide assessment knowledge (p=0.02) and attitudes towards intervention (p=0.01); both were maintained at T3  -Self-rated suicide risk knowledge and applied knowledge did not change significantly from T1 – T2 or T3 (p= 0.22)  -Training evaluated by participants as highly relevant and effective  -52% plan to change their treatment approach, 89% to change their communication  -Training was highly relevant and met objectives |
| Condron et al. (2019)  USA | Quasi-experimental  The study aimed to examine whether differences in training outcomes were observed for brief versus in-depth gatekeeper trainings for trainees from varied professional settings while controlling for differences in trainee characteristics and community context. | Total = 1,918 Participant numbers: 672 from K-12 education settings; 419 from mental health settings; 827 from community settings   QPR (n = 1,133) ASIST (n = 785) Specific numbers not provided - 78% Female; 41.4 years (average)  ~30% worked in mental health (non-specific); ~24% social workers; ~14% other health professionals (non-specific) | Gatekeeper training:  Question, Persuade,  Refer (QPR)  Applied Suicide Intervention Skills  Training (ASIST)  Gatekeeper training teaches skills for the identification and referral of individuals at risk for suicide | QPR:  1–2-hour training  ASIST:  2-day training | QPR:  Designed to teach gatekeepers emergency response skills to identify and intervene with someone at risk for suicide and how to get help for an individual at risk for suicide  ASIST:   - In-depth intervention strategies - Understanding cultural values of suicide   Psychological first aid | Follow-up (3 months)  *-Training Utilization and Preservation*  *Survey (TUP-S)*  TUP-S:  (1) identification of youth at risk  for suicide  (2) referral to services following identification (3) referral to acute services following identification. | -Across the 3 settings a higher percentage of participants who received the ASIST training (in-depth) were able to:   1. identify at risk youths 2. Refer to services 3. Refer to acute services   *When compared with QPR training recipients  -Social workers and mental health professionals performed significantly better (p = 0.001)  *The results suggest that in-depth gatekeeper trainings modify gatekeeper behavior significantly compared with brief trainings. |
| Cramer et al. (2019)  USA | Case Series  We aimed to conduct one of the first evaluations of the effectiveness of a web-mediated core competency-based suicide prevention training program for health professionals. | 239 participants - 43 completed evaluation (18% response rate) 1 Nursing assistant (2.3%); 29 Psychologists (67.4%); 1 Psychiatrist (2.3%); 1 Social worker (2.3%); 11 Counsellors (25.6%).   -36 Females (83.7%)  -36.7 years (average age) | Competency-based:  Training designed by author, with content and training modules based on materials from prior core competency training studies (updated to include more recent guidelines & practices) | Online delivery – self-directed  *20 hours total, completed within 30 days  -Consisted of narrated PowerPoints, handouts, and associated resources | Non-specific:  10 competencies -   1. Manage attitude & reaction toward client 2. Develop/maintain empathy & collaboration 3. Know/elicit evidence-based risk/protective factors 4. Focus on current suicide plan/ideation 5. Determine risk level 6. Develop collaborative treatment plan 7. Notify/involve relevant partners 8. Document risk, plan, and clinical reasoning 9. Know the laws surrounding suicide 10. Engage in debriefing & self-care | Pre; Post (immediate)  -*The Suicide Behavior Attitude Questionnaire*  *(SBAQ)*  -*Attitudes toward*  *Self-Harm Patients Scale (ASHP)*  - Suicide prevention knowledge was assessed using a 15-question multiple-choice knowledge  quiz for pre-post assessment.  -Suicide prevention skill performance was assessed subjectively using *Suicide Competency Assessment Form (SCAF)* and objectively using clinical case vignette. | -Large gains in suicide prevention knowledge & perceived skill ability, capacity to work with suicidal clients and perceived knowledge, ability to assess & manage risk (all p<0.001)  -Moderate gain for perceived ability to help self-harming patients (p=0.70)  -Decrease in negative attitudes towards self-harm (p=0.007)  *Online mediated core competency-based training showed positive impacts on suicide prevention-related knowledge, perceived skill, capacity to work with suicidal patients and reduction of negative feelings about self-harming patients |
| Da Silva Cais et al. (2011)  Brazil | Case Series  This project aimed at improving professionals’ skills in diagnosing and treating mental disturbances that are correlated with suicide as early as possible and at improving health professionals’ attitudes toward individuals with suicidal behavior. The objective of this study was to measure the impact of this training in terms of changing the knowledge and attitudes of the health teams toward suicidal behavior. | 270 health care professionals (only 135 completed both surveys)  *Training was aimed at professionals who are routinely involved with those at high risk for suicide (mostly primary caregivers)  -physicians (57); -Psychologists (69); -OT's (43); -Other: social workers, nurses, etc. (101)  - 229 Females (84.8%)  -Highest number of participants were aged 30-40 years (n=107; 39.6%) | Skills-based:  Suicide prevention training designed for study | Suicide prevention training consisted of 18 hours divided into 14 hours of theoretical exposition and 4 hours of discussion, during which the participants shared clinical cases that they were conducting | Non-specific:  Training aimed at improving knowledge and attitudes in -  -suicide epidemiology (in Brazil & globally)  -general vision of suicidal behavior (historical & cultural influences)  -health team attitudes (attitude construct & impact)  -mental disturbances and suicide (causes & risk factors)  - suicide risk evaluation (steps in risk assessment)  -suicide prevention strategies (recommendations by WHO, national programs, global evidence) | Pre; Post (immediate)  - *Suicide Behavior Attitude Questionnaire*  *(SBAQ)*  3 of the SBAQ subscales were considered for analysis:  1.suicide right  2.negative attitudes towards patient  3.perceived professional capacity for management  *Suicide Prevention Knowledge*  *Questionnaire (SPKQ)* | -Statistically significant increases in SP knowledge (p <0.001); and attitudes across domains (i) suicide right (p= 0.02), (ii) negative attitudes (p=0.002) and (iii) professional capacity (p< 0.001)  *The changes detected by the Knowledge Questionnaire were improved management of depressive patients, the ability to evaluate suicide risk, epidemiological information, knowledge of suicide prevention strategies and ability to evaluate lethal intentionality.  *The training led to improvement in knowledge and attitudes for all ages and occupations despite prior knowledge |
| Donald et al. (2010)  AUS | Case Series  To compare a standard training model for suicide prevention to an enhanced training model.  Enhanced training includes teaching/encouragement to develop organizational links for the purpose of suicide prevention | Public health workers training received: n=242; standard + enhanced training: n=55 Evaluated: n= 111 (45.9% participation); standard + enhanced n= 31 (56.4% participation)  Participants included 92 Nurses (38.3%), 5 psychiatrists (2.1%), 9 doctors (3.8%), 47 psychologists (19.6%), 44 social workers (18.2%), 18 OT's (7.4%) and 25 other (10.4%).  -Highest number of participants were aged 40-49 years (n=91; 38.1%)  -Other demographics not specified | Non-specific - compared two types:  Standard - mixed methods training including videos, exercises, discussion, and didactic group sessions.  Enhanced - didactic and interactive sessions | Standard training – 1 day in person workshop  Versus  Enhanced training – additional 3-day intensive workshops | Non-specific:  Standard: (1) knowledge of effective suicide prevention strategies, (2) expansion of organizational networks, and (3) the expansion of networks against their purpose.  Enhanced: sociological influences and cultural context, recognizing signs, crisis intervention and postvention, relevant therapies, train-the-trainer principles - each participant received a hard & digital copy of the training to encourage sharing of information within the local health district. | Pre; Post (immediate); Follow-up (3 months)  Evaluation measures included  (1) knowledge of effective suicide prevention strategies  (2) expansion of organizational networks  (3) the expansion of networks against their purpose.  25 item questionnaire focused on  (1) recognizing the signs of suicide  (2) crises intervention strategies  (3) brief counselling techniques  (4) postvention strategies. | -Standard training produced increase in knowledge at T2 & T3; however, enhanced training produced statistically significant increase in knowledge (p= 0.001)  -Enhanced training produced significant increases in expansion of organization networks across domains (i) information exchange (p<0.001), (ii) liaison & support (p= 0.01) and (iii) local planning (p=0.001)  *Findings from this study suggest that training efforts that embed a process for implementing and maintaining changes in practice following the initial training event have a greater impact than training that does not provide at least some period of support to staff once they return to the field. |
| Duvivier. L (2017)  USA | Randomized Controlled Trial (RCT)   1. Develop an online training course on youth suicide risk assessment 2. Use an RCT to evaluate the delivery/role of online training 3. Help determine which learners benefit from online training   Examine gains made from online training | Mental health professionals and students - n= 163 (80 Group 1; 83 Group 2) included: (numbers not specified)  - 118 completed evaluations (51 G1; 67 G2) - 45 participants dropped out (27.6%)  *Professionals* - 37% social workers; 39.7% mental health counsellors/therapists; 9.7% clinical psychologists; 1.9% psychiatrists; 11.7% other  - 92% Females  - Ages not provided | Skill-based:  “Tree of Life” training | Self-directed online training modules to be complete within 2 weeks of access.  Group 1 (OLT) completed training upon consent; group 2 (WL) was asked to wait 2 weeks (completed training after T2) | Based on the *Decision Tree Model* of suicide risk assessment, 10 modules:  1.Introduction  2.The Decision Tree Model  3. Acquired capability  4.Desire & Ideation  5. Plans & Preparation  6.Perceived burdensomeness  7. Thwarted belongingness  8. Other risk factors  9.Putting it all together  10. Beyond the assessment | Pre; Post (immediate)  Measures included demographic & training, risk assessment knowledge & skill, satisfaction with training  -*Computer-Based Training Attitudes Scale (CBTAS)*  -*Motivated Strategies for Learning Questionnaire (MSLQ)*  -Study specific 20 item questionnaire – Suicide Risk Assessment Knowledge  -Suicide Risk Assessment Skill - study specific: 36 applied behavioral questions  -Training satisfaction – 8 questions (5-point Likert scale) | -OLT participants scored significantly higher in 'Skill in Determining overall Risk' (p <0.05)  -OLT participants scored higher overall in all domains (knowledge, skill, risk assessment, rating risk factors, self-efficacy), however no difference was seen in 'Attitudes' between the groups (post-training).  -Overall, participants were satisfied with the content & delivery of the training, except one who had difficulty navigating the platform  -Prior suicide prevention training and/or experience working with suicidal individuals did not moderate the learning effect across domains for the OLT group (very small effect) |
| Gask et al. (2019)  Scotland | Qualitative  Designed to disseminate the delivery and implementation of STORM training | Total = 60 study participants **demographics not specified   Nursing - 32 (53.3%)  Psychology - 4 (6.6%)  Occupational Therapy - 5 (8.3%) Physicians - 3 (5%) Other 16 (26.6%) | Skills-based:  Skills Training On Risk Management (STORM) model  -designed for clinical staff to improve attitudes and skills | Training comprises brief lectures,  video  demonstration  and discussion, role-rehearsal, and video-feedback to acquire new skills.  Training for facilitators is carried out over 2 days, following a 2-day experience of being trained in the intervention (total duration of 4 days). | STORM training consists  of up to 4 educational modules which can be delivered  flexibly (but usually over 2–3 h each).  1.Assessment  2. Safety planning (crisis management)  3.Problem solving  4. Future safety planning (crisis prevention) | Follow-up (1-2 years)  Semi-structured telephone interviews –  Facilitators & Managers:  -how was STORM received and what policy/practice/culture changes occurred?  -concerns relating to the delivery of training.  -appropriate implementation?  -is the training targeted at correct population?  -what role did you play in implementation?  Participants:  -experience and view of training?  -impact on everyday practice? | Interview responses:  -implementing training is difficult when not mandated  -Facilitators gained confidence and reassurance from working in pairs and seeking supervision to manage problems  -both facilitators & participants noted positive effect on clinical practice post-training  -training improved participants confidence in asking patients about suicidal behaviors and their ability to identify risks |
| Gomez et al. (2012)  Chile | Case Series  The objective of this study was to investigate changes in attitudes toward suicide among a group of primary health care professionals  that attended a training program in early detection and prevention of suicide risk. | Eighty-nine frontline health professionals: Psychologists (n=52), OT (n=1), Social workers (n=17), physiotherapist (n=1), paramedics (n=1), nurses (3), doctors (n=12) & psychiatrist (n=1))  -70.8% Females  -31.7 years | Skill-based:  Training in early detection and prevention of suicide risk | 2 day in-person training including presentations, analysis of clinical vignettes, small group discussion and training through dramatization techniques. | Non-specific:  Training focused on suicidality, risk and protective factors, indicators of suicide crisis, suicide and life cycle, interview techniques with suicidal patients, evaluation of suicide ideation and suicidal attempt, therapeutic setting, management of risk, therapeutic alliance, work with the family, and CBT | Pre; Post (immediate)  *Suicide Behavior*  *Attitudes Questionnaire (SBAQ)*  *Attitudinal Beliefs Questionnaire about Suicidal Behavior (CCCS-18)*  Study specific instrument based on clinical vignettes | -Overall, the three subscales of the SBAQ showed significant changes. There was a decrease in scores on the subscale “feelings towards the patient” (p = .003), an increase in “professional capacity” (p = .000) and a reduction in “right to suicide” (p = .000).  -Significant changes were obtained in the four subscales of the CCCS-18, with reductions in “legitimation of suicide” (p = .001), “suicide among terminally ill” (p = .000), “moral dimension of suicide” (p = .000) and suicide itself (p = .000).  -positive attitudinal changes and a decrease of negative feelings toward the patient and an increase in the perception of professional competence. |
| Gryglewicz et al. (2017)  USA | Case Series  The aim of this study was to evaluate whether MHPs’ knowledge, attitudes, perceived social norms, and perceived behavioral control in working  with at-risk suicidal youth improve following an online training | 225 mental health professionals were trained – 178 completed the study  -141 Females (79.2%)  -Ages not provided | Gatekeeper:  QPRT: Question, Persuade, Refer, Treat | The online  Training format includes video lectures, audio files, clinical  illustrations, and interactive practice challenges and  quizzes. Completion time for the training is approximately  8–12 hr.  Participants had 4 weeks to complete the training | The training content of QPRT focuses on the epidemiology  of suicide, suicide risk factors and risk management,  engagement and interviewing skills, and continuity of care strategies.  The main component of the training focuses on  teaching skills to assess and manage suicide risk through the use of a structured interview protocol known as the QPRT Suicide Risk Management Inventory | Pre; Post (immediate)  Training outcomes were measured using a 45-item, self-report questionnaire assessing suicide prevention literacy, attitudes, social norms, and PBC relative to engaging an at-risk suicidal youth, and training satisfaction, engagement, and cultural competency.  -Knowledge – 17 multiple choice questions  -Attitudes: 5 item Likert scale (5-point)  -social norms: 6 item Likert scale (5-point)  -perceived behavioral control (PBC): 3 item Likert scale  -training satisfaction: 6 item Likert scale & open-ended feedback  -Cultural competence: 6 item Likert scale | -significant increase in total knowledge (p<0.001)  - large effects seen in risk judgement, crisis management, risk/protective factors (p<0.001)  -significant increase in PBC including confidence (p<0.001)  -overall satisfaction with training and perceived ability to integrate knowledge into practice  -77% of participants rated training as culturally competent |
| Gryglewicz et al. (2020)  USA | Case Series  This study examines the effect of role-play training on MHPs’ attitudes, subjective norms and perceived behavioral control surrounding suicide risk assessment behaviors. | 203 mental health professionals completed training; 178 responded to survey - community mental health, school-based mental health, medical, administration (actual professions not specified)  -79% Females  -Ages not provided | Role-play training | 4.5-hour role-play training following online suicide risk assessment training  (QPRT training from Gryglewicz et al. 2017)  *Within 4 weeks of online training | Non-specific:  Role play training was based on a training curriculum guided by therapeutic alliance principals, behavior modification techniques and engagement strategies  -training content consisted of a review of suicide warning signs, risk and protective factors, suicide terminology, and risk formulation using assessment protocols.  -engagement skills for suicide risk assessment were modelled (building rapport, identifying risk/protective factors, communicating in non-judgmental and empathetic manner) | Pre; Post (immediate)  A 28-item, 5-point Likert scale was used to measure Theory of Planned Behavior constructs known to be strongly correlated with behavioral intentions and behavior change.  1.Attitudes (4 items; perceived importance of engaging in risk assessment/management)  2.Subjective norms (6 items; perceived view of colleague's abilities)  3.Perceived behavioral control (PBC) (18 items; perceived confidence in one's abilities)  A 15 item, 5-point Likert scale measured participants perceptions of training and trainer at post-test; trainer competence, alliance with the trainer, and utility. | - 40% increase in subjective norms and 67% increase in PBC (including determining suicide risk, documenting risk assessment & formulating a safety plan).  -97% of participants felt the trainers were attentive and 85% were comfortable participating in role-play.  -Almost 94% of participants reported their rapport/trust of the trainer helped reduce any role-play anxiety/fear.  -99% reported the training as valuable and skill enhancing. |
| Hawgood, et al.  2022  Australia | Case series  This study examined the impact of STARS training workshop on clinician competencies and analyzed the association between clinician characteristics and pre-training competencies in suicide risk assessment.  It also aimed to determine whether clinician characteristics would predict STARS training outcomes. | 222 participants completed pre-training survey -  144 completed post-training survey (64.9% response rate)  78.1% allied and mental health professionals.  21.9% human services professionals (e.g., welfare worker)  75.7% female  Most aged between 35-44 (34.1%), followed by 45-54 (26.8%). | Competency-based.  Systematic Tailored Assessment for Responding to Suicidality  (STARS)-2 protocol, introduced in 2018.  Developed by lead author.  Adapted from the original 1-day, non-mandatory STARS training protocol, developed in 2015.  Adapted to include design input and co-facilitation by people with lived experience of suicide. | 2 day face to face workshops  Includes six modules:  Module 1- Lived experience and worker attitudes  Module 2- essential concepts in suicide risk assessment  Module 3- structure and application of STARS protocol (Parts A, B and C)  Module 4- Documentation and duty of care  Module 5- Safety planning  Module 6- self-care (and impacts on the worker) | Utilizes structured professional judgement approach (Cramer & Kapusta, 2017), which guides professionals to explore multiple factors to inform care decisions.  Based on competencies by Cramer et al. (2015):   1. Manage attitude and reactions towards client 2. Develop and maintain a collaborative, empathic stance towards client 3. Know and elicit evidence-based risk/protective factors 4. Focus on current plan and intent of suicidal ideation 5. Determine level of risk 6. Develop and enact a collaborative evidence-based treatment plan 7. Notify and involve other persons 8. Document risk, plan, and reasoning for clinical decisions 9. Know the law concerning suicide 10. Engage in debriefing and self-care | Pre; Post (48 hours post training-reminders sent 2 weeks following workshop)  Attitudes to Suicide Prevention Scale (ASP)  Perceived Capability Scale  Declarative Knowledge Scale  Reluctance to Intervene Scale  Social Desirability Response Set (control variable) | Associations between clinician characteristics and pre-training competencies:  - Negative attitudes to suicide prevention had significant but weak correlations with number of fears about conducting suicide risk assessment.  - People with higher perceived capability had higher formal and informal training.  - People with prior experience of client suicide or attempts had greater perceived capability, fewer fears regarding suicide risk assessment, and more declarative knowledge.  - Females had lower levels of reluctance to intervene.  Change in competencies (pre/post)  - Significant changes in all outcomes were found, except for reluctance.  Predictors of change  - Change in attitudes was predicted by amount of previous informal training. Those with more previous formal training reported less attitudinal change. They also reported less improvement in capability.  - Change in declarative knowledge was predicted by gender, with those who reported their gender as ‘other’ reporting greater change in declarative knowledge compared to females. |
| Huh et al. 2012  USA | Case Series  To develop and deliver an educational program to increase awareness and improve suicide risk assessment and management training for a range of health care providers who may see older adults in their care settings. | 132 participants from VA healthcare:  - 67 respondents (51%) to post test -51 respondents (39%) to follow-up  Social workers (58) Psychologists (31)  Nursing (24) Doctors (12) Psychiatrists (9)  Other (5) (included OT’s)  **Other demographics not provided | Non-specific  Designed by authors | 6.5-hour in person workshops (2 hospitals in California) | Non-specific:  Training delivered by presenter to group aimed at improving awareness and attitudes towards suicide risk assessment and management for older adults  -older adults more likely to use lethal methods  -increased risk factors such as chronic disease/pain, social isolation & loneliness  - distinction between dynamic and static risk factors | Pre; Post; Follow-up (3 months)  -Attitudes and Knowledge: self-report 7-point scale  -Clinical behavior assessment: case notes reflecting assessment & management plan based on vignette | - increase in self-perceived confidence in risk assessment & management (sustained at T3)  - improvement in all participants’ overall medical record documentation quality conceptual clarity regarding static versus dynamic risk  -43% of participants reported incorporating skills/knowledge into clinical practice at T3  -90% reported maintenance of increased awareness at T3 |
| Jacobson et al 2012  USA | Case Series  To assess mental health professional’s confidence, attitudes and practice behaviors in suicide risk assessment and management following RRSR training, and if further training was sought after by participants. | 452 mental health professionals: -194 completed all 3 evaluations counsellors (n=118; 26%), social workers (n=100, 22%), psychologists (n=79, 17%), nurses (n=77; 16%), psychiatrists/doctors (n=11, 3%), students/interns (21, 5%), other (79, 17%).  *347 Females (78%)  *43.1 years (average age) | Skills-based:  Recognizing and Responding  to Suicide Risk (RRSR) | Online training module to be completed prior to attending a 2-day skill-based training workshop | RRSR:  Online module - attitudes and approaches to working with those at risk of suicide (with test at the end)  Workshop - 24 core competencies: focused on translating knowledge into practice through the use of extensive guided case application exercises.  Participants learn specific strategies to formulate suicide risk based on the presence of acute risk factors in the context of elevated chronic risk. Participants are encouraged to consider alternatives to hospitalization and increase the use of safety plans  Participants were taught the use of the IS PATH WARM acronym and CASE approach to risk assessment | Pre; Post; Follow-up (4 months)  *Attitudes to Suicide Prevention scale (ASP)*  *Counseling Self-Estimate Inventory (COSE)* - subscales 'Process' and 'Difficult Client Behaviors' only  *Suicide Behavior Attitude Questionnaire (SBAQ) -* 4 items of subscale 'Professional Capacity'  Clinical Risk Management Scale (study specific) - 9 item (4-point Likert) - measured self-confidence in management of clinical risk  Additional questions were modified from the STORM survey to assess - confidence (3 Q's); frequency of skill use (3 Q's); practice behaviors (13 Q's).  Immediate Suicide Risk Management Scale (study specific)  Participants were also asked to assess and respond to 3 detailed vignettes | -Statistically significant results for mean differences in SBAQ scores; 'Clinical Risk Management scale' and Confidence ratings for the modified STORM (p < .001) across time and maintained (T3)  -statistically significant decrease in negative behaviors between timepoints (p< 0.001)  - 'Assessment of Suicide Intent' scores were significant (p= 0.02)  - statistically significant increase in perceived effectiveness of suicide safety plans (p= 0.01)  - statistically significant effect (positive) found from vignette analysis (p=0.02)  -91% of participants reported training implementation at T3  - participants’ skills to assess acute and chronic suicide risk factors and protective factors, formulate risk, and plan a dispositional response to that level of formulated risk increased at T3  - use of online training prior to workshop deemed useful as participants entered the workshop with a baseline level of knowledge |
| Johnson et al. 2011  USA | Case Series  The objective of this study was to evaluate the ‘‘CALM’’ (Counseling on Access to Lethal Means) training, in which community-based mental health care providers were trained to work with at-risk clients and their families to assess and reduce access to lethal means of suicide, including firearms. | Community mental health professionals (individual professions not specified)  196 workshop participants - 168 completed T1; 111 completed T2  -81.4% Females  -42.8 years (average) | Means restriction:  Counselling on Access to Lethal Means (CALM) | 2-hour workshop | CALM:  Instruction about: 1) what lethal means are, 2) how reducing access to lethal means can prevent suicide, and 3) how to conduct a lethal means assessment with a family member of a client.  Training included - a formal presentation (background data, descriptions & justifications for lethal means reduction and overview); introduction to firearms; video re-enactment; instruction & discussion of lethal means assessment conduct; role-play for participants | Post; Follow-up (6-8 weeks)  Study specific questionnaire  5-point scale  -questionnaire evaluated how much participants gained from the workshop, as well as their attitudes, beliefs, and behavioral intentions about counselling on access to lethal means prior to and immediately after the workshop. | - 86% agreed they received concrete ideas for counselling  - 89% agreed they would discuss means reduction in the future  -84% believed CALM met an important need  - results indicate an increase in knowledge and skill regarding lethal means reduction counselling and were maintained at T2  -65% (72 out of 111) reported they had counselled on lethal means since completing the workshop  - limitations include: no baseline data, lack of objective data, attention bias and lack of evaluation of provider by client pre/post training |
| Jones et al. 2018  AUS | Qualitative  The aim of this paper was to report on a formative dialogical evaluation that explored the views of health and human services workers regarding a suicide prevention training program in regional (including rural and remote areas) South Australia which included meaningful involvement of a person with lived experience in the development and delivery of the training. | 248 training participants - 24 interviewed for study (across 8 regional communities)  Human services (n=5), OT's (n=2), School counsellors (n=2), Social workers (n=8), support officers (n=1), other (n=6)  -45.5 years (average age)  -other demographics not provided | Study-specific:  Regional suicide prevention training | 1-day workshops (6.5 hours) in person, delivered by multi-disciplinary team including persons with lived experience | Non-specific: Training topics included -Introduction to suicide  - Aboriginal and Torres Strait Islander Considerations  - Lived Experience Reflections -Myths and Facts  -Reflective discussion  -Engaging with young people (video activity) - Pathways to care in different practice settings - Postvention - what to do following a suicide death  - Supports and Self-care  - Summary of key learning  - Engaging with a suicidal person | Follow-up (~3 months)  1-1 interviews following the training  - each interview lasting ~30minutes  -open-ended questions to help understand the impact and application of the training in clinical practice | -participants expressed strong appreciation of the lived experience component of the training reporting that a lived experience perspective may help to reduce stigma.  -The training empowered them to work with at risk clients (increased confidence)  -Participants reported implementing the training skills  -Participants reported the importance of location/regional area specific training  -Suggestions were made to increase training duration and offer training annually |
| Kawashima et al. 2020  Japan | Case Series  This study evaluated the effect of an assertive-case-management training program. In the present study, we focused on changes in attitude and self-efficacy toward suicide prevention, attitudes toward suicide, and suicide intervention skills. | 322 attended the training; 274 participants completed the evaluations  doctors (n=87), nurses (n=79), social workers (n= 70), clinical psychologists (n= 32), other (public health worker, social welfare officer, n= 6)   -131 Males (48%); 143 Females (52%) -38 years (average age) | Assertive case-management:  Developed by Japanese research team – based on previous studies | The program ran for 2 days (16 hours) and consisted of lectures, group workshops, and role-play practice sessions. Delivered at 10 centers across Japan by multi-disciplinary teams (doctors, social workers, psychologists) | Assertive-case management training included:  -Lectures (evidence-based suicide prevention, relation between psychiatric disorders and suicide, communication, case management [for intervention & follow-up], psychoeducation, bereaved family members, interprofessional collaboration)  -Group work (identifying risk factors, incident response and follow-up)  - Role-play  - Discussions | Pre; Post (immediate)  Japanese versions of the:  *Attitudes to Suicide Prevention Scale (ASP)*  *Gatekeeper Self-Efficacy Scale (GKSES)*  *Suicide Intervention Response Inventory (SIRI)*  *Attitudes Toward Suicide Questionnaire (ATTS)*  *Additionally, a visual analogue scale to measure satisfaction with training. | -Significant increases in Attitudes to Suicide Prevention Scale; Gatekeeper Self-Efficacy Scale; SIRI-1 and SIRI-2 (all p < 0.001)  -effect of training (in attitude and self-efficacy) was highest in those who had no prior suicide prevention training/no experience working with at risk persons.  - training had a higher impact on males than females, which was consistent with SIRI scores from previous studies |
| La Guardia et al. 2019  USA | Case Series  Evaluates a competency-based suicide prevention training | 38 attended training; 29 completed evaluation: Counsellors (n=20), psychologists (n=1), social workers (n = 2), school counsellors (n=2), substance/alcohol counsellor (n=3), nurses (n=1), other (n=1)  -26 Females (89.7%) -41.3 years (average age) | Competency-based:  Zero Suicide Model - multi-level framework  *10 competencies:  1. Manage attitude & reactions  2. Develop/maintain empathy & collaboration  3. Know/elicit evidence-based risk/protective factors  4. Focus on current suicide plan/ideation  5. Determine risk level  6. Develop treatment plan  7. Notify/involve relevant partners  8. Documentation  9. Legal knowledge  10. Engage in debriefing & self-care | In-person half-day workshop, supplemental handouts provided as take-home resources | Addresses clinical care and systems/ administrative suicide prevention strategies.  The content featured factual; research based  content for each core competency, supplemented by a series of case studies, discussion points, and self-reflective exercises to allow for feasible and accessible practitioner training in the community | Pre; Post (immediate)  Suicide Risk Assessment and Management  Knowledge – 12 item multiple-choice quiz based on training content  *Suicide Competency Assessment Form (SCAF)*  *Suicide Behavior Attitude Questionnaire (SBAQ)* – only 2 subscales (1. Feelings towards client, & 2. Professional capacity)  *Attitudes toward Self-Harm Patients (ASHP)* – 15 items  *Interprofessional Socialization and Valuing Scale (ISVS)* – 9 items evaluating participants attitudes towards interprofessional education (only administered at T1) | Statistically significant results found for -  Knowledge (increased correct answers on multiple choice quiz) p <0.001  Competency (SCAF scores improved) p =0.001  Perceived ability to help patients (improved ASHP scores) p < 0.001  Improved Optimism and Patience (ASHP) p = 0.04  Confidence and Adequacy of training (AHSP) p= 0.008  Professional Capacity (SBAQ) p < 0.001  *'Negative feelings towards patients' (SBAQ) was improved but not significantly (p = 0.71)  -Evidence of a connection between interprofessional socialization and perceived self-efficacy of working with suicidal individuals. |
| Levitt et al. 2011  USA | Case Series  This study evaluated  a suicide awareness and prevention training workshop for providers  working with mentally ill homeless and previously homeless adults. | 120 trainees – social workers and paraprofessionals (not specified)  60 completed pre-test, 120 completed post-test, 53 completed follow-up   -86% Females  -Ages not provided | Study specific: Gatekeeper “type” training  Based on the *Practice*  *Guideline for the Assessment and Treatment of Patients with Suicidal Behaviors* (American Psychiatric Association, 2003) | Half-day in person training workshop – PowerPoint presentation and training manual | Non-specific:  Training content designed to assist participants to recognize indicators of and risk factors for suicidality, obtain and coordinate  care for suicidal clients, document this work appropriately, and communicate effectively  with co-workers and external service providers concerning these issues.  Primarily didactic training (limited interactive elements) | Pre; Post; Follow-up (21 months)  Pre & post - 23 item multiple choice test, which covered material on both epidemiology  and prevention, with roughly half the test  items addressing each area (specific domains not provided).  21-month follow-up was conducted for 53 participants (able to be contacted) | Posttest scores were markedly improved  over pretest scores (p <0.001)  -Follow up scores were slightly lower than post-test scores, but remains substantially higher than pre-test scores (indicating maintenance of knowledge)  -Overall, training was effective in increasing the suicidality- related  knowledge of both professional and paraprofessional  mental health service providers, as demonstrated by both subjective and objective measures, and this benefit was sustained over time. |
| Matthieu et al. 2014  USA | Case Series  The primary objective of this study was to evaluate a suicide prevention  training program and secondarily, to conduct an educational needs assessment of gatekeepers working in community hospice settings. | 39 health staff working in community hospice care  Counsellors (n=3); Social workers (n= 32); Other (n=4)   -35 Females (89.7%) -43.1 years (average age) | Gatekeeper training:  Question, Persuade, Refer (QPR) | 1–2-hour on-site training. QPR training includes a lecture, 10-minute video, a question and answer  period, referral cards, and concludes with a behavioral rehearsal component  tested | QPR training:   - designed to teach gatekeepers emergency response skills to identify and intervene with someone at risk for suicide and how to get help for an individual at risk for suicide | Pre; Post (immediate)  Training specific measures:  -Perceived self-efficacy (10 items)  -Declarative knowledge (14 items – multiple choice)  -Satisfaction and impact of training (4 items -5-point Likert)  -Awareness of resources and referrals (4 items- dichotomous response options)  -Behavioral rehearsal session was evaluated using a 10-item Role Play Acceptability Scale (4-point Likert) | Training increased awareness of the risk factors for suicide, as reported by nearly 80% of participants.  Self-efficacy scores showed a statistically significant increase (p = 0.00)  Satisfaction and the need for additional devoted time for suicide prevention  training was highly rated.  -1/5 of participants reported knowledge of suicide prevention (SP) efforts in their workplace  -Less than ¼ reported knowledge of local & state SP efforts |
| Midorikawa et al. 2020  Japan | Case Series  We aimed to investigate the association between general mental health and attitudes toward suicide of participants in suicide-prevention gatekeeper training programs. | 230 participants – 115 completed questionnaires   Doctors, nurses, social workers, and non-medical professionals (numbers not specified) - medical workers (n=71; 61.7%), non-medical workers (n=44; 38.3%) -87 Females (75.7%)  -Ages dispersed evenly across age groups; highest number of participants aged 60-69(n=27;23.5 %) | Gatekeeper:  Non-specific – designed for study | Half-day workshops | Non-specific  The training consisted of lectures about  basic knowledge for suicide prevention (60 min), role-play and group work applying the TALK steps (Tell, Ask, Listen and KeepSafe) with  original teaching videos we created (90 min), and a Q&A session  (30 min). | Pre; Post (immediate)  *Demographic information  *Prior training experience  Japanese versions of - *General Health Questionnaire (GHQ-12)* – 12 items  *Attitudes Towards Suicide (ATTS)* – 37 items | 27 of the 115 respondents (23.5%) were classified as having poor mental health  -Results suggest that those with poorer mental health have increased ‘permissive attitudes’ towards suicide (when compared to good mental health group)  - perceiving suicide as ‘unavoidable’ may be problematic for intervention  Gatekeepers with poor mental health may influence their own counter-suicide behavior/intervention. |
| Mirick et al 2016  USA | Case Series  This study examined the impact of a 1-day continuing education training for mental health professionals on knowledge and confidence around suicide assessment and intervention. | 543 participants - 442 completed pre and post-tests:  social workers (n=162), mental health counsellors (n=116), psychologists (n=76), nurses (n=32), students and other (including OT's) (n=57); majority of participants worked in mental health (n=360)   -330 Females (80.9%)  -Ages not provided | Competency-based:  Best Practices in Suicide Assessment and Intervention called  *Suicide Assessment and Intervention Training (SAIT)* | 6-hour in person CPD workshop - led by instructors who are both mental health professionals and content experts in the field of suicide. Instructors use PowerPoint presentations, practice exercises (including role plays both by participants and trainers) which include opportunities for behavioral rehearsal, short videos, individual writing activities and discussions. | *Suicide Assessment and Intervention Training (SAIT)*:  #1 - Understanding suicide  #2 – Risk/protective factors  #3 – Managing reactions  #4 - Therapeutic empathy  #5 - Eliciting the suicidal narrative  #6 – Risk assessment tools  #7 - Formulation of risk  #8 - Crisis intervention and safety planning  #9 - Best practices in treatment (overview)  #10 - Standard of care  #11- Suicide postvention  #12 - Self-care | Pre; Post (immediate)  25 item questionnaire (7-point Likert) measuring knowledge and confidence  -pre-test also included 'demographics' and 'work experience' questions (including prior SP training, years of clinical practice, working with suicidal individuals) | -Prior SP training and/or experience moderated learning (scored higher)  -Participants reported that the knowledge they gained regarding 'safety planning', 'assessment tools', 'validity techniques' and 'asking about suicidal behavior' were both new skills and the most valuable parts of the training.  -all participants demonstrated increased knowledge and confidence post-training, regardless of prior experience/ training (indicating efficacy of SP training)  - results of study suggest that CPD training in suicide prevention is highly useful to increase knowledge and confidence |
| Mirick et al 2020  USA | Qualitative  This qualitative  research study interviewed participants of a one-day continuing education training titled *Suicide Assessment and*  *Intervention Training for Mental Health Professionals* to explore how it changed their clinical practice | 60 participants – social workers (n= 19), mental health counsellors (n=17), psychologists (n=12) and other (n=12)  -49 Females (81.7%)  -Ages not provided | Competency-based:  *Suicide Assessment and Intervention Training (SAIT)*is a 6-hour workshop with 12 modules (see Mirick, 2016) | 15–20-minute interviews were audio-recorded and transcribed verbatim | Participants had attended the SAIT training up to 2 years prior  (See Mirick, 2016) | Follow-up (1-2years)  Semi-structured interviews:  Are there things you do differently or think about differently when you assess for suicidal thoughts and behaviors as a result of the training  you attended? If so, what have you changed?  Can you give an example of a time when you used the material you learned in the training?  Have you noticed any changes in the ways clients have responded to you as a result of these changes? If so, what are these changes? | 90% reported they had changed their practice post-training; the 3 main themes to these changes include –  1.converstations with clients changed (increased comfort and improved approach to questions)  2.management of clients (use of best practice in safety planning and improved ability to control own emotions)  3.stronger relationships with clients (responding more positively to suicidal behaviors) |
| Painter et al. 2018 | Case Series  A training program for pharmacists was used to provide skills necessary to recognize a crisis and the warning signs of suicide. The program's effect on the participant's general perception, self-efficacy, and attitude towards suicide prevention was examined. | 103 pharmacists were trained, 77 responded to survey  -41 Females (53.2 %) -43 years (average age) | Gatekeeper training:  Based on Question, Persuade, and Refer (QPR) program targeting pharmacists. | 1.5-hour workshops delivered across 4 locations | The training was divided into 3 parts: (1) importance of  pharmacists to be trained in suicide prevention, medications  as a common method of suicide, and ways for pharmacists to  detect those at high risk for suicide; (2) suicide statistics  including protective and risk factors; and (3) Question,  Persuade, and Refer (QPR) training, including role-play exercises. | Pre; Post (immediate)  Study specific  Program Outcome Evaluation - Items included demographics, general perception, self-efficacy, and attitude toward suicide prevention.  General perception - 10 items  Self-efficacy - 7 items  Attitudes - 4 items  Also, 'Demographics' and 3 questions regarding 'bias toward suicide prevention' | -There was no statistically significant difference in respondents' general perception of SP  - more than 75% of participants reported increased confidence in identifying signs, respond appropriately, give reassurance, provide resources, and make referrals  -Statistically significant changes in attitudes toward suicide prevention were observed (p < 0.0001)  - significant effects on changing attitudes in updating knowledge and making appropriate interventions |
| Pisani et al. 2012 | Case Series  To test whether an innovative, brief workshop can improve provider knowledge, confidence, and written risk assessment in a multidisciplinary sample of ambulatory and acute services professionals and trainees. | 338 participants - counsellors (n=76), psychologists (n=51), psychiatrists (n=40), nurses (n=77), social workers (n=75), other (n=19)  -266 Females (78.7%)  -41.8 years (average age) | Competency-based:  Commitment to Living:  Understanding and Responding to Suicide Risk (CTL).  Content was delivered through visual concept mapping and teaches core competencies in assessment and safety planning almost entirely through medical record documentation examples and exercises, also CTL provides context-specific options for responding to suicide risk. | 3-hour in person workshop divided into 2 modules (Assessing Suicide Risk and Responding to Suicide Risk), each module comprised a lecture (20 min) interspersed with case-based discussions (45 min) and a documentation exercise (20 min). | Non-specific - designed by author to with the aim of creating a practical and authentic skill-building experience for clinicians.  It targeted 9 of the 24 competencies for mental health professionals outlined by the American Psychiatric Association of Suicidology across 4 key areas:  1.Attitudes and approach  2.Understanding suicide and collecting assessment information  3.Formulating risk  4.Developing a treatment and services plan | Pre; Post (immediate)  Study specific:  Provider Confidence (Pre/Post - Self-report): 9 items (8-point Likert) that evaluated of confidence in assessing and managing risk for suicide  Knowledge (Pre/Post - Multiple Choice Test): 13 items - knowledge of suicide risk assessment and management.  Documentation of Risk Formulation (Pre/Post - Objective rating): participants completed documentation exercise of suicide risk from a clinical vignette  Perception of Potential Transfer of Training (Post Only Self-Report): 18 items | -Participants’ knowledge and confidence scores improved significantly (p < .001)  -The mean rating of overall quality of participants’ documentation sample increased from 3.1 to 4.2.  The PTT scores indicated that participants perceived that they had sufficient knowledge, motivation, and resources to implement the educational content in their workplace.  The diverse sample of participants showed large improvements on all self-report and objective measures, with no significant differences in change scores among participants from acute, ambulatory, and other services. |
| Pullen, J  2016  USA | Case Series  The quality improvement project included two elements: an evidence-based suicide prevention gatekeeper training program for personnel, and a depression and suicide risk screening instrument for newly admitted residents. | 43 participants - Nursing and allied health personnel: nurses (n=28), social workers (n=2), physical therapists (n=2), OT's (n=1), SP's (n=1), OT aides (n=2), other (n=7)   -39 Females (91%)  -44.8 years (average age) | Gatekeeper training:  Question, Persuade, Refer (QPR) | 2-hour workshop with take-home resources | The Question-Persuade-Refer (QPR) Suicide Prevention Gatekeeper Training is a three-step program on how to ask about suicidal thoughts and feelings, how to persuade the person to seek help, and how to refer to appropriate resources | Pre; Post (immediate)  9-item self-evaluative survey: on knowledge, understanding, resources  *Attitudes Toward Suicide Prevention Scale (ATSPS)* 14 items | The self-report evaluations showed improved knowledge in identifying those at risk and increased awareness of risk factors, participants also reposted increased confidence and "comfort" in asking clients about suicidal thoughts/behaviors.  Participants reported they now felt identifying and managing those at risk for suicide was within their domain of professional responsibility and increased comfort in therapeutic communication |
| Scott, M. 2021  USA | Mixed-Methods:  To evaluate the integration of cognitive behavioral therapy (CBT) for depression and suicide prevention (CBT-SP) into social work practice with youth | Purposive sample of 22 social workers - Participants represented clinicians from public and private agencies, private practice, hospital settings, and all areas of the targeted region.  -14 Females (63.6%)  -48.3 years (average age) | Skills-based:  Cognitive Behavioral Therapy for Suicide Prevention (CBT-SP) | A 2-day training (two 6-hr day trainings including didactic lecture, videos, role plays, and discussions) was supplemented by reading material, website support, and 3 months of weekly group telephone consultation | Training Day 1:  -Components of CBT for depression (general CBT knowledge, cognitive reframing, behavioral activation)  Training Day 2:  -safety planning  -components of CBT for suicide prevention  Group clinical consultation was provided by telephone weekly for 3 months with a qualified team clinician to discuss and process barriers to utilization of the CBT-SP techniques. | Pre; Post; Follow-up (3 months)  Knowledge of CBT and CBT-SP - included 26 items comprised of both true–false and open-ended questions about the specific CBT-general (12 items) and CBT-SP (14 items) techniques and the purpose of the techniques.  Demographic and clinical practice survey - demographics and prior experience  Barrier to Implementation of Evidence-Based Practice Scale - 15 items (5-point Likert) evaluating perceived individual and organizational barriers to implement EBP. | -Significant increase in knowledge of CBT (general) and CBT-SP skills and knowledge (all p < 0.000).  -There was no difference in the change in knowledge by characteristics of the participants including gender, age, and prior CBT experience  -participants reported the skills they were most likely to use/integrate into practice were "chain analysis" and "safety planning"  -all participants reported learning new techniques and 95% reported integrating them into their practice at the 3-month follow-up |
| Serra-Taylor et al. 2016  Puerto Rico | Case Series  The aim of the study was to design and evaluate a training program for health professionals in universities in Puerto Rico. Training focused on the development of skills and knowledge in the evaluation and management of suicide risk in university students. | 39 professionals in counselling (64.9%), psychology (27%) and social work (8.1%).  -82.1% females  -Ages not provided | Study specific  Suicide prevention training designed for counselling and social work staff working in university settings | 1-day face-to-face workshop (8h) | Non-specific:  Training content focused on: key competences according to the American Association of Suicidology, statistics on suicide, importance of screening, ethical-legal aspects, risk and protective factors, evaluation of risk, professional approach, the Columbia-Suicide Severity Rating Scale (C-SSRS), determination of level of risk, suicide risk management, safety plan or prevention agreement, documentation, evidence-based therapies for the management  of suicidal ideation and behavior, and postvention. | Pre; Post (immediate)  20-item survey measuring intervention skills (e.g. evaluation of hypothetical cases) and knowledge (e.g. risk factors, definitions, statistical data and evidence-based therapies) | Statistically significant differences were found between pre and post-tests for (a) knowledge relating to the percentage of suicidal individuals who present mental health disorders, (b) knowledge about the definition of suicide attempt according to the C-SSRS, (c) knowledge about the therapy that has greatest empirical evidence for the management of ideation and suicidal behavior, (d) skills around identifying self-injurious behavior without suicidal intent, and (e) skills related to when evaluate suicidal ideation and behavior (all p = .000)  -There was a 28.7% increase in knowledge and 12.3% increase in skills pre-post, with an overall increase of 20.5% across all survey questions.  Results suggest that an evidence-based training that follows the core competencies and includes practical exercises, increases the knowledge and intervention skills of mental health professionals in the assessment and management of suicide risk. |
| Shim et al. 2010 | Case Series  An educational program was designed for emergency department personnel, piloted with a convenience sample of clinicians, and preliminarily evaluated with particular attention to increasing knowledge and enhancing self-efficacy | A convenience sample of 54 clinicians:  nurses (n=27), social workers (n=13), nurse specialists (n=4), other (n=10)  -46 Females (85.2%)  -43.7 years (average age) | Study specific  Suicide prevention training designed for emergency personnel - Participants were given specific advice on how to manage patients deemed to be at high risk and how best to de-escalate crises. | 2 h of didactic lectures, and  1 h of participant discussion | Non-specific -  Managing Suicidality in the Emergency Department:  Four main areas -  1. statistics and definitions pertaining to suicidality  2. risk and protective factor identification  3. lethal means restriction  4. management of acutely suicidal patients in the emergency department.  Participants were also taught 'The Basic Suicide Assessment Five-step Evaluation (B-SAFE) Protocol' which includes:  (1) Identify risk factors  (2) Identify protective factors  (3) Ask specifically about suicide, suicidal ideation, and history of suicidal behavior  (4) Determine risk level and appropriate interventions,  (5) Document the assessment, treatment plan, and instructions. | Pre; Post (immediate)  28 item surveys consisting of 16 multiple choice questions surrounding knowledge and 12 items (5-point Likert) scale regarding self-efficacy  Additionally, there were 4 items that evaluated the participants attitudes towards the training (5-point Likert)  •How helpful was the training on Managing Suicidality in the Emergency Department that you just participated in?  • How relevant was the training that you just participated in?  • How likely are you to use the information presented in this training in your work?  • How likely would you be to recommend this training to others? | Pre-test and post-test measures showed an increase in knowledge and self-efficacy scores regarding management of suicidality in the emergency department immediately after participating in the training session.  Findings revealed a statistically significant increase in knowledge scores from pre- to-post-test (p <0.001) where mean scores increased from 7.9 to 13.6 (out of 16)  and similarly for the self-efficacy component of the survey (p <0.001) where mean scores increased from 24 to 32.3 (out of 48)  - 89.5% rated the training as very or extremely helpful & 84.2% as extremely relevant  -86.9% reported being "very likely" to use the training content |
| Slovak et al. 2019  USA | Mixed Methods - Case series and Qualitative  A concurrent mixed methods approach was used to explore 1) geriatric case managers’ attitudes, beliefs, and behavioral intentions about counselling on access to lethal means following CALM training, and 2)  perceived barriers to assessing suicidality and counselling clients on access to firearms. | 70 participants attended the training, only 60 completed the surveys.  5 of the 60 participated in the focus group portion of the study   *3 most common degrees were nursing (n=28; 47%), Social work (n=18; 30%) or Other (n= 8; 13 %) *58 Females (97%)  *43.5 years (average age)  *Focus group = 4 females, 1 male; 41 years (average age) | Means restriction  Counseling on Access to  Lethal Means (CALM) training | 5-h workshop - components 1&1 delivered in didactic manner, component 3 (CALM) | 3 main components to the training:  1. Understanding suicide risk among older adults  2.Assessing for suicide risk using QPR  3. Counselling on access to lethal means (CALM component)  CALM training covers the following:  (1) background on suicide data and lethal means,  (2) an introduction to firearm suicide data,  (3) video presentation that models the counselling strategy,  (4) a presentation and discussion on conducting a counselling session  (5) role plays | Post; Follow-up (3 & 5 months)  Quantitative survey data collected at posttest and follow-up. Additionally, a focus group and open-ended question in the follow-up survey provided qualitative data for analysis. | -25% of participants reported having never receiving any formal suicide prevention training, and 92% reported never receiving training in counselling about firearms  -70-80% of participants reported the training- gave them concrete skills to use in their work; met an important need, will help them counsel on lethal means, addressed an aspect of suicide prevention that is often overlooked and was relevant to their work.  -many of these results showed a statistically significant increase at follow-up (5 items were p<0.05)  - Focus group reported that the training increased their knowledge about firearm access, signs and symptoms of suicidality and acquisition of skills and strategies |
| Stallman et al. 2019  AUS | Case Series  The aims of this study are to:  (a) evaluate the effectiveness of the Care.Collaborate.Connect online suicide prevention training program in improving knowledge, attitudes, self-care, and confidence in working with people with suicidality in both experienced health professionals and students in health disciplines and (b) evaluate the acceptability of the training program to participants. | 303 health professionals and students from disciplines - psychology (44.9%), nursing (20.1%), social work (14.2%), counselling (8.6%) occupational therapy (4.0%), and medicine (3%).  -85.8% Females  -34.8 years (average age) | Needs- and Strengths-based:  Care.Collaborate.Connect approach - The needs of the client disclosing suicidality are: (a) to be listened to and understood, (b) to be supported to use healthy coping strategies, (c) to connect with additional temporary professional supports as needed. | Self-directed 8-module e-training program that takes about 8 hours to complete. | Care.Collaborate.Connect: Suicide Prevention training:  This approach not only focuses on suicide, but on suicidal behaviors and other stigmatized coping strategies (including non-suicidal self-harm, alcohol, or drug use).  8 modules:  1.Self-care - psychoeducation  2.Attending to distress  3.Talking about suicide  4.Coping planning  5. Problem solving  6.Documentation  7. Ethics and Law  8. Self-management | Pre; Post (immediate)  *The Suicide Prevention Training Evaluation Tool (SPTET)* was used (except for the competency subscale as this was not required for study), which evaluated:  -Knowledge (15 items, multiple choice & T/F)  -Attitudes (15 items, 4-point Likert)  -Confidence (7 items, 4-point Likert)  -Self-care (6 items, 4-point Likert)  -Training satisfaction (5-items, 4-point Likert) and an optional open-ended comments question after the training satisfaction questionnaire. | Participants showed significant improvements in knowledge, attitudes, confidence, and self-care pre- to post-training (all p<0.001) with moderate to very large effect sizes.  -There was a very high level of training satisfaction from participants. Almost all participants agreed or strongly agreed that the training improved their knowledge of suicide prevention (97.1), improved their skills (98.7%), improved their confidence (97.4%), and improved their understanding of the relationship between coping strategies and suicide (99.1%).  - The online format enabled participants to learn at their own pace |
| Stuber et al.  2023  USA | Case series  This study examined the impact of a suicide training program on health professionals’ knowledge, attitudes and confidence regarding suicide prevention. | Health care professionals from Washington State  1548 completed pretraining survey; 873 completed post training survey (56% response rate).  Only 873 responses from pre and post were included in analysis.  Average age 45  66% female  30% non-white  53% Doctors of Medicine, 11% registered nurses, 9% advanced registered nurse practitioners | All Patients Safe.  This is a large-scale training course developed by the University of Washington to help health care professionals meet the state’s 6 h suicide training requirement. | 6 hours, online course with self-paced modules | Non-specific  Based on adult learning principles, with opportunities for case-based, interactive learning. e.g. videos of professionals modelling appropriate care. | Pre; post  All Patients Safe survey- 21 items | - Statistically significant improvements in knowledge, attitudes and confidence occurred in 20 of the 21 items.  - Knowledge statistically improvements in all 4 knowledge questions.  - Attitudes also significantly improve post training, especially with regards to attitudes about conversing about firearms and medication storage.  - There was also a significant increase for all 8 items measuring confidence in applying suicide prevention skills. |
| Terpstra et al. 2018  Netherlands | Case Series  (1) determine the short-term effectiveness of the gatekeeper training program on individual participants  (2) ascertain whether the training has different outcomes for different employment sectors. | 526 professionals attended training sessions - 502 completed pre-training survey, only 174 completed post-training survey  Employment sectors (post-training survey) - Education (n=49), health care (n=70), socioeconomic (n=32), other (n=23)  *Other demographics not provided | Gatekeep training:  Based on QPR but tailored for application in the Netherlands | 4-hour workshop | Based on QPR It consists of four main parts:  (i) introduction - topic of suicide  (ii) theoretical background - epidemiology, risk factors, talking about suicide  (iii) role-plays - practiced 3 times for increased learning/consolidation  (iv) referral pathways - discussion | Pre; Post (6 weeks)  Self-report questionnaire - adapted from QPR evaluation  10 items covering demographics (3 items), knowledge (4 items) and confidence (3 items) | -There were significant improvements in professionals’ knowledge and confidence (p < 0.001)  - the training provided significant increases in knowledge and confidence to address suicide regardless of employment sector. |
| Wharff et al. 2014  USA | Quasi-experimental  To evaluate the provision and effectiveness of brief suicide assessment and management training within the social work department of a hospital | Hospital social work staff - 110 participants, 72 survey respondents  -Demographic information not provided | Competency-based  Study specific - Suicide risk assessment training  Based on eight of the 10 skill-based core competencies for assessment and management of individuals at risk for suicide  -All participants were trained in the *Suicide Assessment Five-Step Evaluation and Triage (SAFE-T)* procedure. | In-service training consisted of two 1.5-hour sessions held one month apart - didactic and discussion/practice-oriented | Non-specific:  First Session -  -Culturally competent suicide assessment  -De-escalation techniques  -Safety planning  -Assessment and inquiry  -Risk/protective factors  -Documentation  -Resources for hospital consultation  -Review of hospital emergency responses  *Second session* -  Consisted of small-group discussions focusing on clinical applications of the eight competencies covered in the didactic session. | Follow-up (3 months)  Evaluation survey - 25 items assessing attendance, understanding, perceived competence, quality, and utility of training. | 97.8% of respondents reported that all domains covered in the training were either helpful or very helpful.  100.0% reported positive changes in perceived competence in 6 of 8 suicide assessment and management skill-based domains.  The mean score on the nine-questions relating to understanding of content was 91.4%. |
